# Eastern Mediterranean Mobility in the Bronze and Early Iron Ages: Inferences from Ancient DNA of Pigs and Cattle

**DOI:** 10.1038/s41598-017-00701-y

**Published:** 2017-04-06

**Authors:** Meirav Meiri, Philipp W. Stockhammer, Nimrod Marom, Guy Bar-Oz, Lidar Sapir-Hen, Peggy Morgenstern, Stella Macheridis, Baruch Rosen, Dorothée Huchon, Joseph Maran, Israel Finkelstein

**Affiliations:** 1 Institute of Archaeology, Tel Aviv University, Tel Aviv, 69978 Israel; 2 The Steinhardt Museum of Natural History, Israel National Center for Biodiversity Studies, Tel Aviv University, Tel Aviv, 69978 Israel; 3 Institute for Pre- and Protohistoric Archaeology and Archaeology of the Roman Provinces, Ludwig-Maximilians-University Munich, Schellingstraße 12, 80799 München, Germany; 4 Zinman Institute of Archaeology, University of Haifa, Mount Carmel, Haifa 31905 Israel; 5 Institute for Prehistory, Protohistory and Near Eastern Archaeology, University of Heidelberg, Marstallhof 4, 69117 Heidelberg, Germany; 6 Department of Archaeology and Ancient History, Lund University, Helgonvägen 3, 223 63 Lund, Sweden; 7 Israel Antiquities Authority, POB 180, Atlit, 30300 Israel; 8 Department of Zoology, Tel Aviv University, Tel Aviv, 69978 Israel

## Abstract

The Late Bronze of the Eastern Mediterranean (1550–1150 BCE) was a period of strong commercial relations and great prosperity, which ended in collapse and migration of groups to the Levant. Here we aim at studying the translocation of cattle and pigs during this period. We sequenced the first ancient mitochondrial and Y chromosome DNA of cattle from Greece and Israel and compared the results with morphometric analysis of the metacarpal in cattle. We also increased previous ancient pig DNA datasets from Israel and extracted the first mitochondrial DNA for samples from Greece. We found that pigs underwent a complex translocation history, with links between Anatolia with southeastern Europe in the Bronze Age, and movement from southeastern Europe to the Levant in the Iron I (ca. 1150–950 BCE). Our genetic data did not indicate movement of cattle between the Aegean region and the southern Levant. We detected the earliest evidence for crossbreeding between taurine and zebu cattle in the Iron IIA (ca. 900 BCE). In light of archaeological and historical evidence on Egyptian imperial domination in the region in the Late Bronze Age, we suggest that Egypt attempted to expand dry farming in the region in a period of severe droughts.

## Introduction

The period between ca. 1,450 and 950 BCE – much of the Late Bronze Age and the early Iron Age – was one of the most dramatic in the history of the Eastern Mediterranean basin^1^. During the Late Bronze Age the great Egyptian and Hittite empires ruled over extensive regions of the Near East and northeastern Africa, Mycenaean palatial societies flourished in what is currently Greece and the shores of western Turkey, and Cyprus functioned as a regional supplier of copper. This was a period of pronounced “globalization”, characterized by strong trade relations that created a cultural *koine*, perhaps best represented by the cargo of the Uluburun shipwreck [on the southern coast of Turkey^2^]. This period of great prosperity ended in a major collapse process known as the “Crisis Years” (refs 1 and 3; for possible relation to climate change see refs 4 and 5). The breakdown of the old order was accompanied by movements of groups, sometimes called the Sea Peoples (among them the Philistines), from various parts of the Mediterranean to the Levant (e.g., ref. 6). These momentous processes shaped the history of the Old World, opening the way to the emergence of new regional kingdoms, among them biblical Israel and Judah.

In the Late Bronze Age, various commodities were shipped along Eastern Mediterranean coasts, including copper ingots from Cyprus, prestige ceramic vessels from the Mycenaean world and Cyprus, and resin from the coast of the Levant (e.g., ref. 2). Most of these items are conspicuous to archaeologists. However, because of the difficulty of detecting migrating animals by traditional research methods, animals and the products processed from their meat have not been sufficiently taken into consideration as have other kinds of mobile goods.

DNA analysis is a powerful tool for studying how humans enabled mobility of animals in antiquity (e.g., refs 7 and 8). Meiri *et al*.^9^ have recently shown that pigs from the Bronze and early Iron Ages in Israel shared haplotypes with modern and ancient Near Eastern pigs, and that European haplotypes appeared in archaeologically-derived pig bones ca. 900 BCE, suggesting a late second millennium date for their first introduction into the region. This raises the possibility that pigs were brought to the region by groups of Sea Peoples, who migrated to the Levant in the Iron I (ca. 1150–950 BCE)^6^. These findings raise questions regarding cultural traditions of migrating groups, decision making of the migrants before boarding the ships and knowledge of the target lands. And they raise broader queries: Were animals routinely transported in the context of the Eastern Mediterranean *koine* of the Late Bronze Age and the collapse which spelled its end? Were other animals brought to the Levant? From where? What can be learned from movement of animals about the mobility of people at that time, and what can be learned about their socio-economic considerations? In the current study we shed light on these issues by presenting new data on ancient pig and cattle DNA in the Eastern Mediterranean basin.

Domestic cattle are abundant at archaeological sites. They are classified as *Bos taurus taurus*, also known as taurine cattle or humpless, and *Bos taurus indicus*, also known as zebu or humped cattle (here they are referred to as taurine and zebu respectively)^10^. Mitochondrial DNA (mtDNA) of domestic cattle shows a phylogeographic structure, which is valuable in tracing human movements (e.g., refs 8, 11 and 12). As humans expanded from the Fertile Crescent throughout most of Eurasia, taurine cattle spread with them. They reached the Aegean with the early farmers in the seventh millennium BCE^13^. Zebu cattle are native to the Indian subcontinent; from there they spread to Iran and Iraq ca. 3,000 BCE^14, 15^. Both sub-species are evident today in Africa. When and how they were brought there is a matter of debate. Some contend that taurine cattle arrived in Africa ca. 7,000–5,000 BCE (e.g., refs 16 and 17). The earliest evidence of zebu in Africa comes from a ca. 2,000 year old site in southwestern Kenya^17^. Genetic studies based on extend samples support the view that introgression of male zebu with female taurine took place once the zebu reached Africa (e.g., refs 18–20). The degree of this introgression is related to movement of humans, and thus makes it another strong marker for studying translocation of cattle^18–20^.

The hybridization of zebu and taurine cattle however is not limited to Africa. Palestinian cattle of the beginning of the 20th century were a small, low producing breed. Later, with increasing modernization, different breeds were imported from other parts of the Middle East and from Europe^21^. Interestingly, this local breed is thought to possess a genetic component of zebu^21, 22^. The time, location and nature of this zebu-taurine introgression remain unknown. Zebu appear intermittently in the iconographic records of second millennium BCE Egypt and the southern Levant (ref. 15 and references therein) and are reported in contemporary archaeozoological assemblages^23^. However, morphological distinction between zebu and taurine cattle is difficult; the only clear indication is the forked dorsal spine of the thoracic vertebrae, which appears in some zebu individuals^24^.

## Research Design

In order to investigate the transfer of animals in the Eastern Mediterranean in the Bronze and early Iron Ages, we took two steps:

1. We broadened our previous study^9^ both chronologically and spatially by extending our pig-database for the Levant and added pig DNA data for two sites in Greece (Fig. 1).

**Figure 1:**
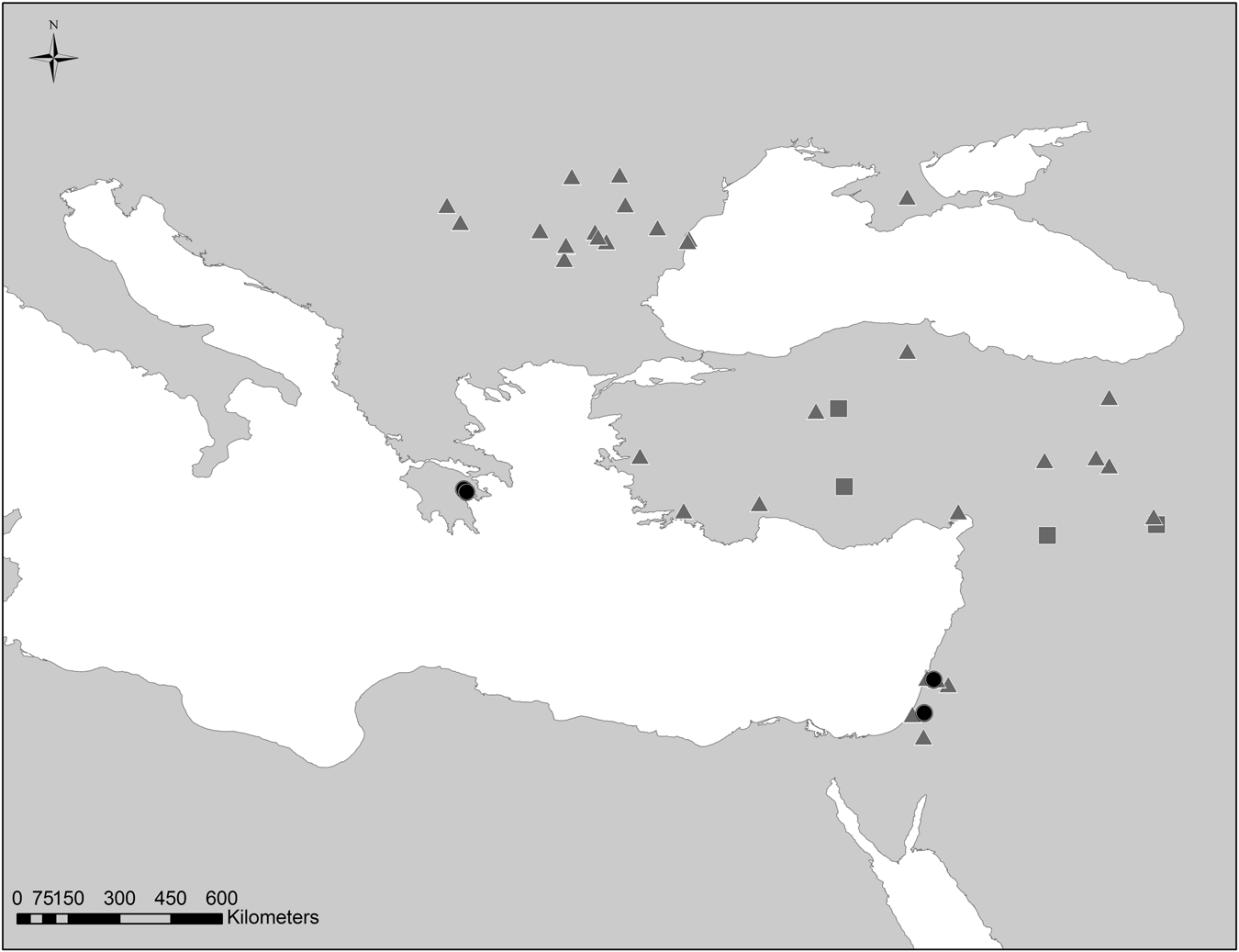
Map showing the archaeological sites which provided pig and cattle samples for this study (black circles), and published ancient data for pigs (grey triangles), and cattle (grey squares). The map was created using the ArcGIS for Desktop (ArcMap 10), ESRI. For additional data see Supplementary Tables S1 and S3.
2. We sampled cattle from four sites in Greece and Israel, providing the first pig ancient mtDNA for samples from Greece and the first cattle ancient mtDNA and Y chromosome nuclear DNA from Greece and Israel.

We used short fragments of the mitochondrial control region (CR) to define the haplotypes of ancient pigs and cattle^11, 25–29^. We also examined single-nucleotide polymorphism (SNPs) cattle Y chromosome^30^ in order to test possible introgression between taurine and zebu cattle. To complement the genetic data, we applied morphometric measurements of cattle metacarpals, as metapodia are the most distinctive bones for distinguishing breeds in ungulates^31^. We compared our measurements of cattle metacarpals from Late Bronze and Iron Age sites in Greece and Israel to Grigson’s dataset^32^ in order to test morphological variability possibly related to sub-genus population differences.

## Results

### Pigs

**Figure 2:**
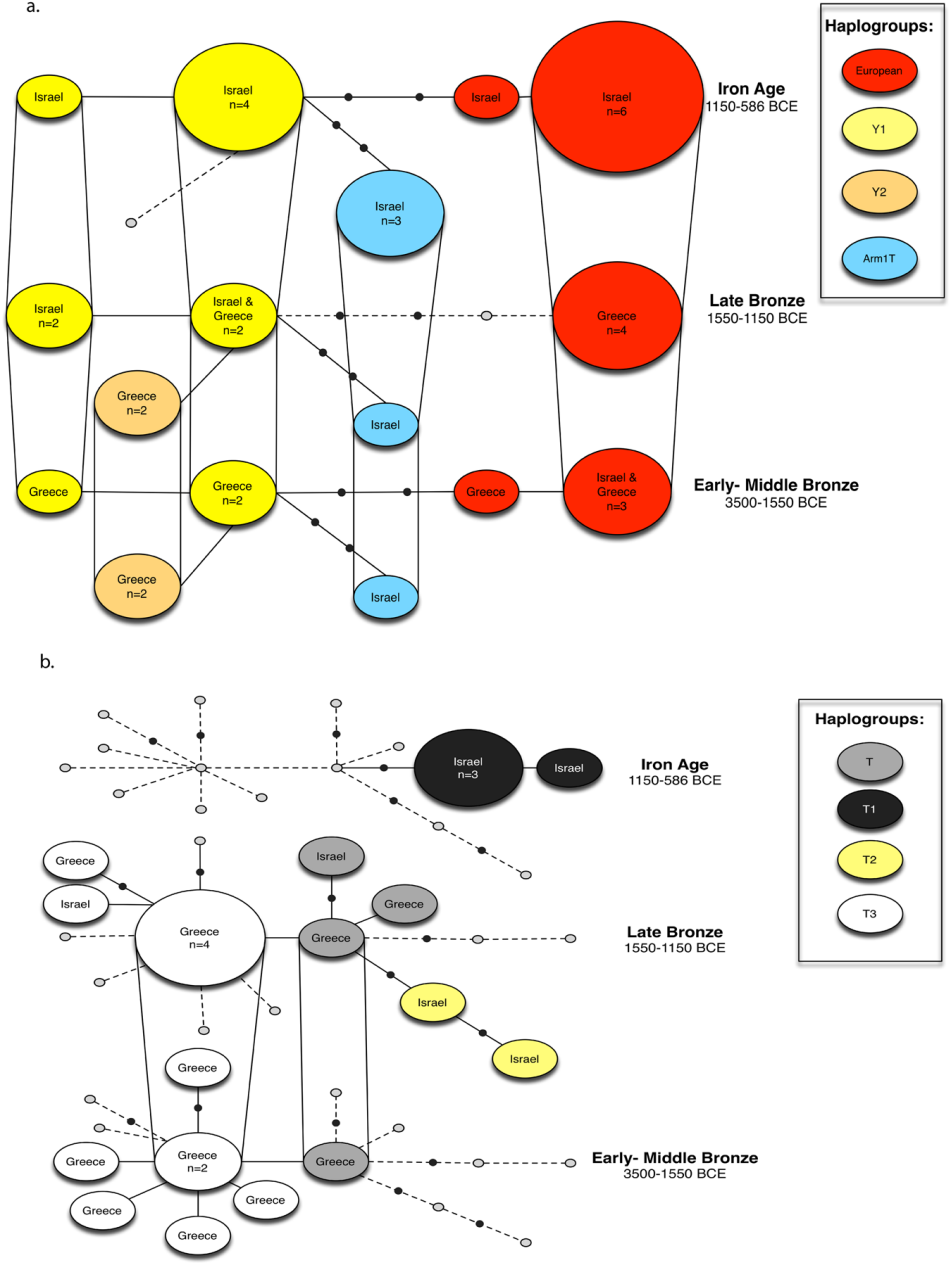
Three-dimensional statistical parsimony network for mtDNA CR of: (**a**) Pigs based on a 76 bp fragment and 36 specimens sequenced in this study. (**b**) Cattle based on a 151 bp fragment and 23 specimens sequenced in this study. Layers represent the haplotypes in three different time periods (Early-Middle Bronze Age, Late Bronze and Iron Age; bottom to top respectively). Each circle represents one haplotype. The colour corresponds to the different haplogroups. Small black dots represent missing haplotypes. The size of the circle is proportional to haplotype frequency. Vertical lines link haplotypes found in both time periods. The networks were created using TempNet^67^ script in R^68^.

**Figure 3:**
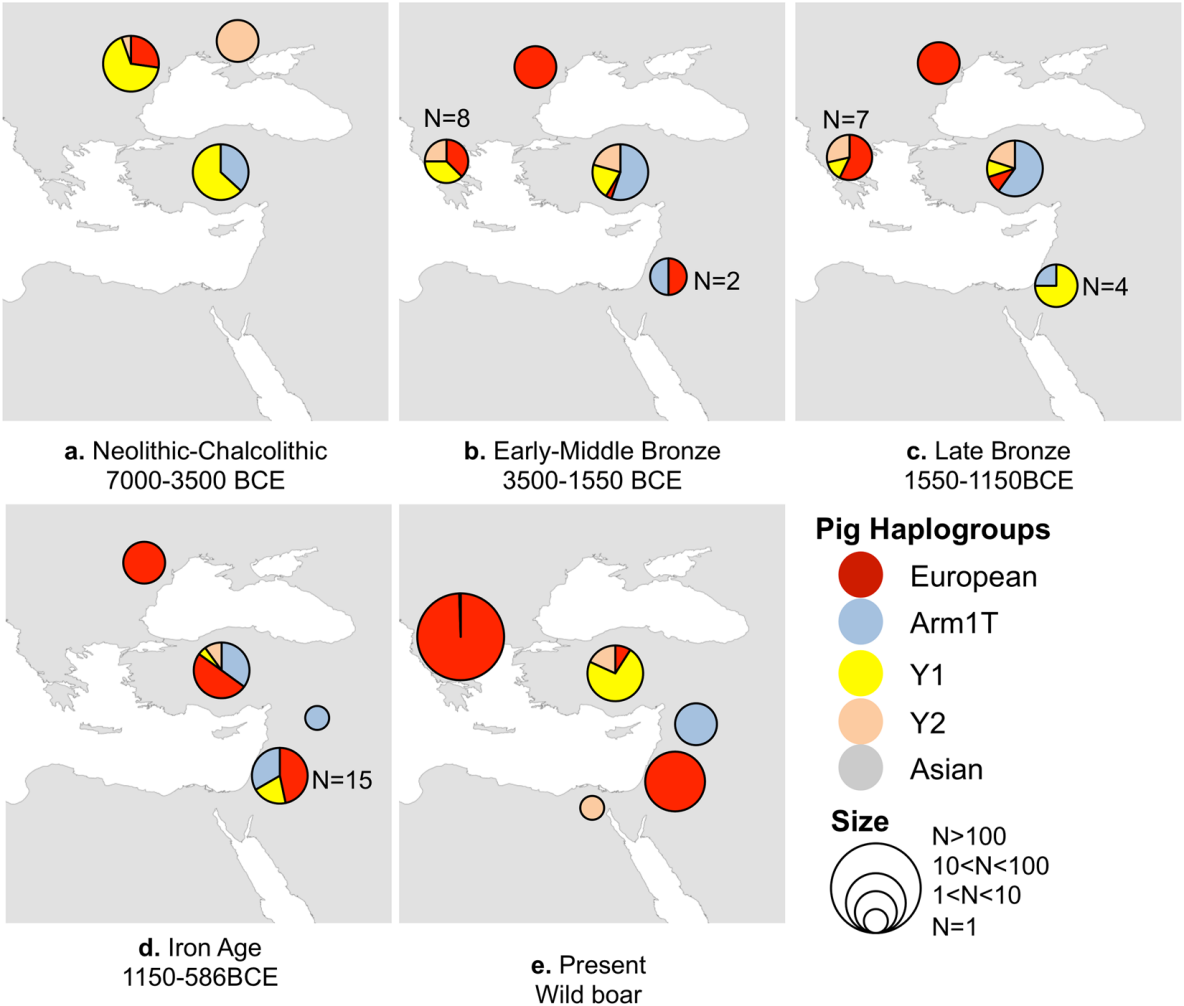
Pig mtDNA haplotype frequency comparison in the Eastern Mediterranean by period. Haplotype names are after Larson *et al*.^25^. The maps were created using the ArcGIS for Desktop (ArcMap 10), ESRI. For additional data see Supplementary Tables S1 and S3.

We successfully extracted and sequenced DNA from 16 out of 52 ancient domestic pig specimens sampled: 15 from Bronze Age Greece and one from Iron Age Israel (Supplementary Table S1). We found four haplotypes in Bronze Age Greece: Y1, Y2 and two European haplotypes (ANC-Aside and ANC-Cside; Supplementary Table S1) (haplotype names are based on terminology of fragment ANC1^25–28^). Three of these haplotypes found in Greek pigs (the two Europeans and Y1) are shared with Israel (Fig. 2a). Haplotypes Y1 and Y2 were commonly considered to be Near Eastern, although recently doubts have been raised regarding the origin of Y2^33–35^; more below). The frequency between the different haplotypes in Greece does not change much between the Early and Late Helladic periods (ca. 3500–1150 BCE, corresponding to the Bronze Age in the Levant) (Fig. 3b and c). The new sample from Israel, dating to the early Iron IIA, carries the European ANC-Cside haplotype. This result conforms to our previous data, which indicate appearance of the European signature of domestic pigs in the southern Levant in the Iron Age, starting ca. 900 BCE, and thus transport of European pigs to the Levant (Fig. 3 and ref. 9).

Haplotype Arm1T has been recorded by us only in Israel (between the Middle Bronze and the Byzantine periods^9^). It is known in Anatolia starting in Neolithic times (Fig. 3a and ref. 28), but was not found in Greece or Western Europe. This implies that haplotype Arm1T can be considered as local Near Eastern/Anatolian, meaning that it did not reach Europe with early Near Eastern farmers^25, 33–35^.

### Cattle

We successfully extracted and sequenced DNA from 25 of the 136 ancient cattle specimens sampled. Ten samples are from Megiddo and Azekah in Israel and 15 are from Asine and Tiryns in Greece. These are the first ancient DNA cattle sequences from Israel and Greece (Supplementary Table S1).

Today taurine cattle have five main haplogroups: T, T1, T2, T3 and T4^11, 29^; T–T3 are found in the Near East. Haplogroup T3 predominates in local European cattle breeds, while T2 can be detected in the Balkans, Italy and sporadically in western Asia. Haplogroup T1 is dominant today in African cattle breeds, and T4 in the East Asian breeds (e.g., refs 11, 12, 29, 36 and 37).

**Figure 4:**
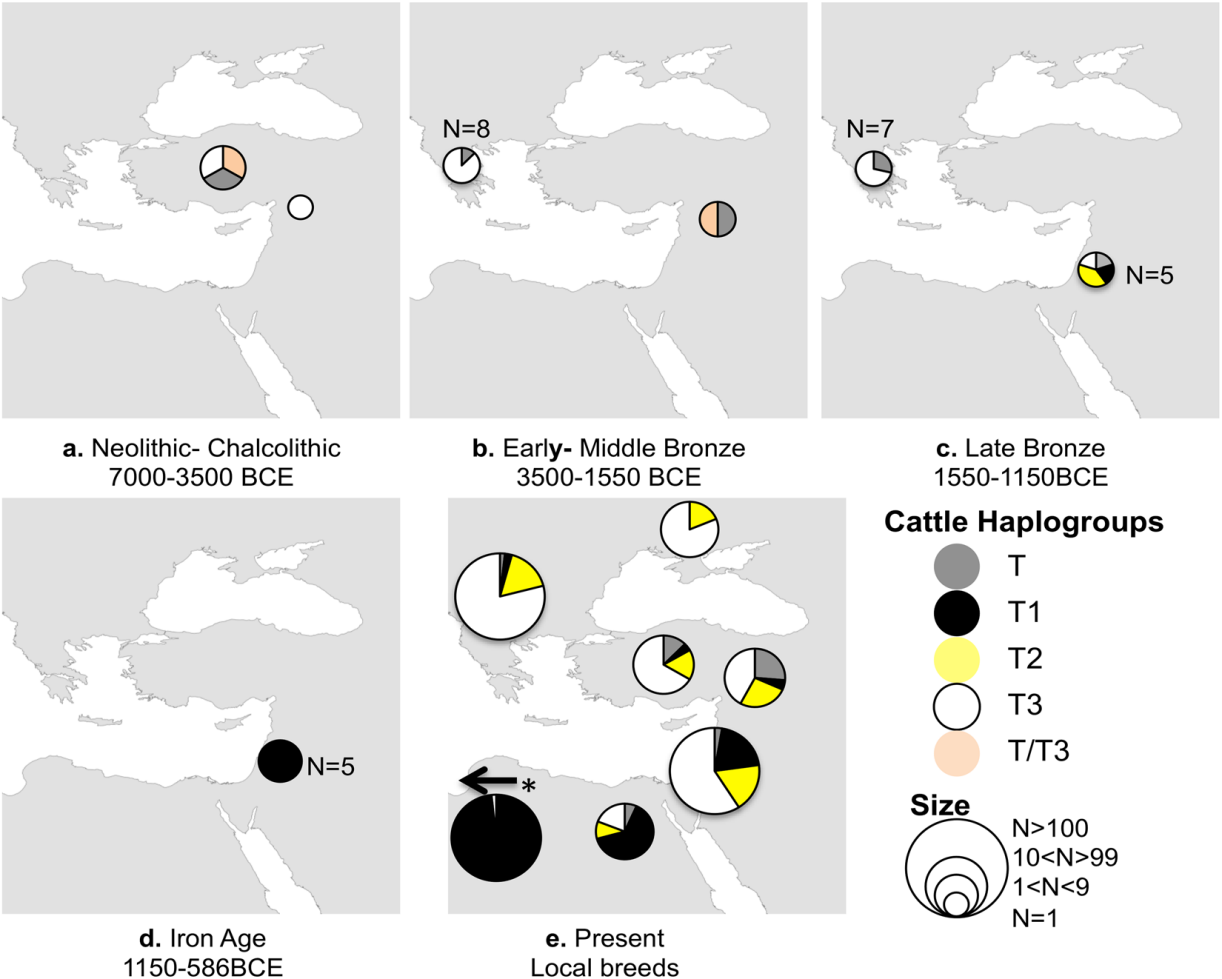
Cattle mtDNA haplotype frequency comparison in the Eastern Mediterranean by period. Haplotype names are after Troy *et al*.^11^. The maps were created using the ArcGIS for Desktop (ArcMap 10), ESRI. For additional data see Supplementary Tables S1 and S3.

For the Bronze Age, the 15 ancient DNA sequences from Greece reveal two haplogroups – T and T3 – with similar frequencies in the Early and Late Bronze Ages (Fig. 4b and c), while in the five samples from Israel four haplogroups are found (T, T1, T2, T3; Fig. 4c). We have no samples from Greece for the Iron Age; in Israel, only haplogroup T1 is found in the five samples that yielded DNA (Fig. 4d).

Although haplogroups T and T3 are present during the Late Bronze Age in both Greece and Israel, the ones from Israel carry a further mutation, meaning that the two regions do not share any haplotype (Fig. 2b). In addition, two samples from Iron Age Israel, with haplogroup T1, carry a mutation at 16,122 base pair position of the mtDNA genome, and are therefore classified as haplotype T1c– a sub-group abundant in modern-day Egypt^37, 38^.

We tested all cattle samples that yielded DNA with three pairs of primers designed to amplify SNPs in the Y-chromosome. In two samples we succeeded to amplify two of the three: one from Early Helladic Asine in Greece (MM506) (3100–2000 BCE) and the other from late Iron IIA Megiddo in Israel (MM422) (ca. 900 BCE) (for the BLAST scores, see Supplementary Table S5). The sample from Asine was identified as taurine cattle (*Bos taurus taurus*), while the one from Megiddo was found to be zebu (*Bos taurus indicus*), meaning that the Greek bull was sired by a taurine, while the Israeli one was sired by a bull with zebu paternal ancestry.

**Figure 5:**
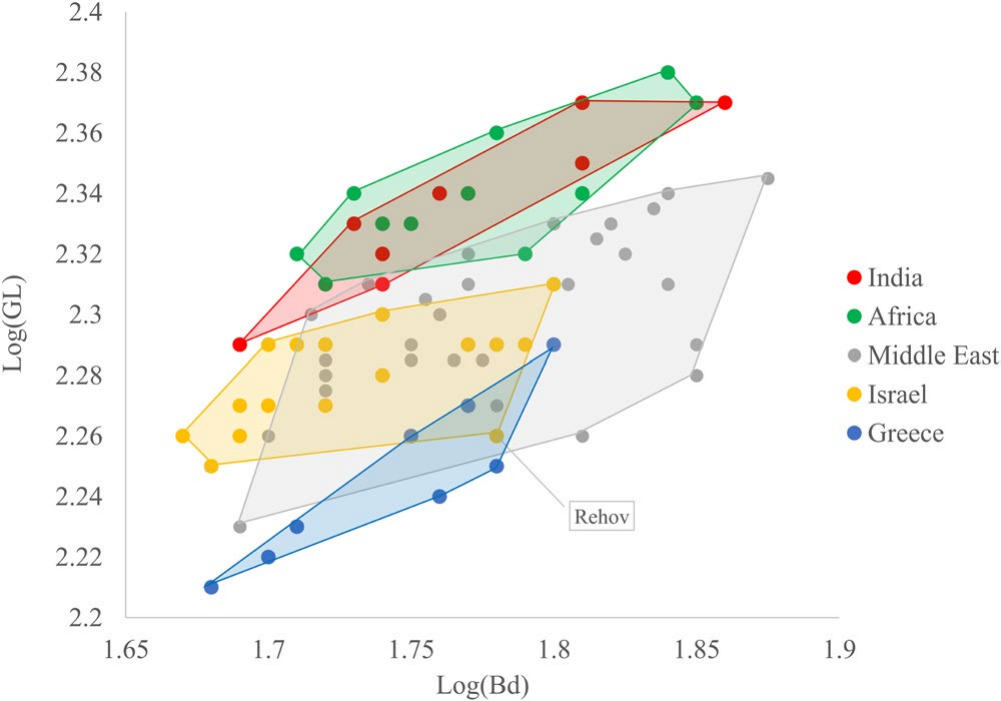
Cattle metacarpus measurements; Greatest Length [log(GL)] against distal Breadth [log(Bd)] (both in mm). Archaeological cattle from India, Africa and the Near East, Late Bronze Age cattle from Greece, and Late Bronze and Iron Ages cattle from Israel (Supplementary Table S4). Convex hulls are the smallest convex polygons containing all points of a respective group.

Complete or nearly complete cattle metacarpal bones are rare finds in second millennium BCE archaeological sites in Greece and Israel, with small sample sizes hindering the use of sophisticated shape-analysis statistics such as Geometric Morphometrics (GMM) and Linear Discriminant function Analysis (LDA)^39^. A more traditional metric analysis was conducted on twenty-eight Late Bronze and Iron Age cattle metacarpals from Greece and Israel, which were measured and compared to Grigson’s dataset (ref. 32 unknown contexts; Supplementary Table S4). The log-transform of the two measurements Great Length (GL) and distal Breadth (Bd) were plotted against each other (Fig. 5).

Zeboid cattle from India and Africa are very similar in terms of metacarpus proportions, and both are easily distinguishable from Late Bronze Age cattle from Greece (Fig. 5). Middle Eastern archaeological cattle are more variable in terms of metacarpus length from both groups (India and Africa vs. Greece), and occupy an intermediate position between them, perhaps suggesting its position as a zone of hybridization. Note that one specimen from Iron Age IIA Tel Rehov in Israel (Fig. 5) classifies with the Late Bronze cattle from Greece, may indicate a rare appearance of European cattle in Israel. Assignment of specimens to known groups was successful in 67% of the cases (Discriminant Analysis, PAST 3.14^40^).

Due to the rarity of assemblages dated to this period and the high level of bone fragmentation which renders GL measurement either impossible^41^ or difficult to come by, we unfortunately could not obtain measurements of earlier cattle metacarpi from the third millennium BCE. Therefore, the morphometric data lack the crucial benchmark of pre-introgression domesticated cattle metapodial shape. Still, the fact that only one (5%) out of 19 specimens from Bronze and Iron Age Israel occupies the European part of the morphospace may suggest that introgression of European cattle morphotypes – if it indeed occurred – was negligible.

## Discussion

We observe different population histories for pigs and cattle during the Bronze and Iron Ages in the southern Levant. Incorporating archaeological and historical data, we suggest two types of translocations to the southern Levant, which are distinct by place of origin of the animals (Aegean region vs. Egypt), and the route taken (sea vs. land).

### Pigs

We have observed the transfer of pigs between Greece and Anatolia at least as early as the third millennium BCE by detecting the Near Eastern haplotype Y1 in Greece in the Early Helladic period (3100–2000 BCE, continuing into the Late Helladic period; Fig. 3). We are aware that Y1 may have been local to Greece much earlier than the third millennium BCE and may represent descendants of pigs brought from the Near East during the Neolithic period^25, 34^. Regrettably, we do not as yet have earlier samples from Greece to verify this issue. Still, we would argue that what we see is the result of connections between Greece and Anatolia (and regions further to the southeast), as archaeological evidence indicates exchange of goods between the two regions as early as the Early Bronze Age^42, 43^.

A recent genetic study of modern pig and wild boar samples shows that the local signature of mainland Greece is composed of specific European haplotypes, which are not found elsewhere in Europe, thus probably representing a relic-population from the Last Glacial Maximum^44^. These results, as well as the presence of haplotype Y1 in Sardinia and Sicily in the Late Bronze Age^45^, emphasize the extent of exchange across the Mediterranean during the Bronze Age.

Haplotype Y2 is considered to have a Near Eastern origin^27, 28^. However, the existence of pig haplotype Y2 in our Greek samples during the Early Helladic II (one radiocarbon determination – 2875–2581 cal BCE) (Fig. 3) together with the findings of Mesolithic wild boar remains in Romania and northeast Italy^33, 35^ challenge this conventional wisdom. The absence of haplotype Y2 from Anatolia in the Neolithic (despite a large sample size, n = 38^28^) on one hand, and its presence in Romania during this period on the other^33^ suggest a west-to-east translocation, from Greece to Anatolia no later than the Early Bronze Age.

Displacement of pigs from Greece to the southern Levant should probably be understood against the background of movement of groups of Sea Peoples to the east. This translocation is attested in ancient DNA samples dating to the early phases of the Iron Age (no later than ca. 900 BCE^9^). The ca. 200–250 year gap between the first arrival of Sea Peoples on the southern coast of the Levant and the first evidence of European pig mtDNA can be explained by the small sampling size, especially taking into account that no pig samples from Philistine sites have thus far yielded DNA^9^.

Pigs are favoured livestock for marine transportation, as they are relatively small and have an omnivorous diet [e.g., refs 46 and 47; it is unlikely that they were brought from Europe by land – they are poor candidates for long-distance herding and almost impossible to drive (e.g., ref. 48)]. Indeed, their translocation goes together with immigration by boat^26, 49^: they reproduce rapidly and thus make it easier to quickly establish herds and produce much meat^50^. A good example is provided by Anglo-Saxon sites in Britain, which show a large percentage of pig remains at the beginning of the settlement process (early 5th century CE). Once the community was established, cattle and sheep consumption increased at the expense of pigs^51^. It seems therefore that at least some groups of Sea Peoples came to the southern Levant aiming to establish settlements^50^.

### Cattle

To differ from the case of the pigs, our genetic data does not indicate movement of cattle between the Aegean region and the southern Levant (no shared haplotypes during the Bronze and Iron Ages; Fig. 2).

The genetic results show an increased frequency of taurine cattle haplogroup T1 (which is dominant in Africa) in Israel in the transition from the Late Bronze to the Iron Age, as well as the appearance of haplotype T1c, which is dominant today in Egypt (refs 37 and 38 and Fig. 4). Since the data for cattle in the Levant is scarce and as there are no samples to represent ancient Egypt, it is impossible to determine the origin of haplotype T1c. Yet, based on archaeological and historical data we suggest that taurine cattle were translocated from Egypt to the southern Levant.

The morphological measurements indicate that Israeli cattle breeds are located between taurine and zebu cattle, which could be the result of interbreeding between the two sub-species (Fig. 5). This observation is supported by the fact that, today, native Near Eastern cattle breeds possess some levels of zebu signature in their nuclear autosomal DNA (e.g., refs 19, 20, 52 and 53). So far we do not have data from pre-second millennium Levantine domestic cattle to check this issue further.

Stronger evidence for the appearance of zebu in the southern Levant is found in our genetic data. The Y-chromosome of one sample from Megiddo (MM422) was identified as zebu. This sample, which comes from a late Iron IIA (9th century BCE) context, carries the taurine mtDNA haplotype T1c, providing the earliest evidence for hybridization between taurine cattle and zebu in the Levant.

It seems that farmers in antiquity were aware of the advantages of crossbreeding taurine cattle with zebu. Especially noteworthy is the better heat tolerance of the zebu (due to low metabolic rates, many large sweat glands and large skin surface), as well as better resistance to insects, ticks and protozoa^16^. It is not surprising, then, that today’s zebu and their crossbreeds dominate in relatively arid regions such as the Indian subcontinent and most of Africa^16^.

Due to poor state of preservation, only two of our samples yielded Y- chromosome DNA. It is therefore impossible to say with certainty at this point whether taurine-zebu crossbreeding occurred in the southern Levant, or if crossbreeds were brought to Canaan from Egypt. For historical reasons we prefer the former option as detailed below.

During much of the Late Bronze Age, ca. 1450–1130 BCE, the southern Levant was under Pharaonic imperial domination, with Egyptian garrisons and bureaucrats stationed in several key-locations, such as Gaza, Jaffa and Beth-Shean near Megiddo^54^. Excavations at the latter two sites revealed evidence of monumental Egyptian construction, and from the Amarna tablets we learn that Egyptian administrators operated in different parts of the region^55^. Egyptian imports – ceramics, stone vessels, seals etc. – are indicated in all major centres in Canaan^56^ and Egyptian impact on the economy of Canaan is attested, for example, in the Amarna letters and in hieratic inscriptions from southern Israel (refs 55 and 57 respectively).

The above-reported results can be understood against this background, bearing in mind that the interest of the Egyptian colonial administration was to enhance and exploit the agricultural output in Canaan, and that an important function of cattle was ploughing. Indeed, archaeozoological investigation of the faunal assemblages from Late Bronze Megiddo demonstrates continuous increase in cattle frequencies during the period of Egyptian rule in Canaan^58^, cattle were kept to older age – an indication of their use as plough animals^59^. Improvement of cattle by crossbreeding between the taurine cattle and the resilient zebu must have been highly advantageous, as it could facilitate the expansion of agriculture, especially dry-farming. This was especially true in view of the evidence of severe droughts at the end of the Late Bronze Age (ca. 1,250–1,100 BCE)^5^.

The complex and dynamic translocations of biological organisms from the Aegean region and Egypt into the Levant during the Bronze and Iron Ages were not restricted to livestock. Frumin *et al*.^60^ present the case for the introduction into the Levant in the Iron Age of new flora, such as cultivated sycamore and opium poppy from Egypt and Aegean region respectively. The sycamore is an east African species that was domesticated in Egypt. The spreading of poppy, the source of opium, is likely to be the result of trade between Greece and other parts of the Eastern Mediterranean. The earliest example for poppy in southern Greece comes from Tiryns of the Late Helladic IIIB2, in the second half of the 13th century BCE^61^.

## Conclusion

The results of this study suggest that human mobility through trade and migration in the Bronze and Iron Ages influenced the composition of livestock in both the southern Levant and Greece. The data presented here shows that each species narrates its own history, depending on its natural characteristics and economic value.

Pigs were translocated between the Aegean region, Anatolia and the southern Levant during the Bronze and Iron Ages. To differ, there is no evidence for movement of cattle across the Mediterranean at that time.

The evidence for crossbreeding between taurine and zebu cattle, together with archaeological and historical data for the involvement of imperial Egypt in the Levant in the Late Bronze Age, seem to shed light on the environmental and economic situation in the region. When evaluated with evidence for increase in cattle frequencies and indications that cattle were kept to older age, the new ancient DNA data seems to reflect attempts by the Egyptian administration to expand dry-farming in the “green” zones of the region in a period of severe droughts – in the later phase of the Late Bronze Age.

## Materials and Methods

### Sample collections for DNA analyses

*Bos* and *Sus* samples were collected from four archaeological sites: Megiddo and Azekah in Israel, and Tiryns and Asine in Greece (Fig. 1 and Supplementary Table S1).

Megiddo in northern Israel is a focal site for the study of the Bronze and early Iron Ages in the Levant. The excavations manifest good control over stratigraphy, pottery assemblages (providing relative chronology) and radiocarbon dating^62^. Samples of pigs and cattle were taken from Early Bronze, Late Bronze and Iron I–II layers.

Tiryns in the northeastern Peloponnese, Greece, is one of the most important sites for the study of the Late Helladic (Late Bronze Age) period in the Aegean Basin. Samples were taken from contexts dating to the late palatial period (Late Helladic IIIB) and the post-palatial period (Late Helladic IIIC) (13th and 12th centuries BCE) from the Lower Citadel and the northeastern Lower Town.

Azekah, a multi-period mound in the Judean lowland in Israel; samples were taken from Late Bronze layers.

Asine in the northeastern Peloponnese, Greece; samples represent the Early Helladic period (third millennium BCE).

For the current study we sampled in total 52 and 136 ancient pig and cattle bones and teeth respectively, spanning the Bronze and Iron Ages (ca. 3500 BCE to 586 BCE) (Supplementary Table S1). The samples are of domestic pigs and cattle as evident from morphometric and osteological data. All samples derive from secure stratigraphic contexts, in many cases with multiple radiocarbon-dates (e.g., refs 62 and 63). Additionally, five pig and five cattle specimens from Asine in Greece were radiocarbon dated [Supplementary Table S1; ref. 64].

### DNA extraction and amplification

DNA extractions and preparations for PCR reactions of the samples were carried out in a dedicated ancient DNA lab, at the Institute of Archaeology of Tel Aviv University, Israel. The lab is located in a building where no molecular work has taken place.

DNA from both pig and cattle bones and teeth was extracted according to a method modified from Yang *et al*.^65^. Specifically, 1 ml of extraction buffer [0.44 M EDTA (pH = 8) (AMRESCO, USA), 0.1 M urea, and 20 mg/ml proteinase K (AMRESCO, USA)] was added to about 50 mg of bone powder and incubated overnight at 56 °C. After decalcification and digestion, the supernatant was concentrated to about 100 μl using Vivaspin filter 3000 MWCO (Sartorius Stedim Biotech), and then directly purified using silica based spin columns (Minelute PCR Purification kit, QIAGEN, Inc).

Short fragments of the mtDNA CR were amplified. For the pig samples, following Larson *et al*.^25^, a short fragment of mtDNA CR was amplified with ANC1F (5′-CTTTAAAACAAAAAAACCCATAAAAA-3′), and ANC1R (5′-TTAATGCACGACGTACATAGG-3′). This 74 bp fragment is highly variable and can distinguish between European, Near Eastern and East Asian pig haplotypes.

For the cattle, depending on the preservation state of the samples, different primer pairs were used to amplify the highly variable region of the mtDNA CR between bases 16,022 and 16,262^66^ (Supplementary Table S2a). In cases of poor preservation, new primers were designed to specifically target the nucleotide positions that define the major four haplotypes. In addition, to test for hybridization between *Bos taurus taurus* and *Bos taurus indicus*, three primer pairs were designed to amplify SNPs in three Y-chromosomal genes: DBY1, DBY7, ZFY4 [following^30^; Supplementary Table S2b].

PCRs and post-PCR work were performed at the Zoology Department of Tel Aviv University, Israel. PCR amplifications were performed in 25 ul reactions with: 1x PCR buffer, 1.5 U of Platinum Taq DNA polymerase High Fidelity, 2 mM MgSO_4_, 0.2 mM of each dNTP (all Invitrogen, UK) 0.1 mg/ml Bovine Serum Albumin (New England, BioLabs, UK)/Rabitt Serum Albumin (Sigma-Aldrich Inc.) for the pigs and the cattle respectively, 0.4 µM of each primer (Sigma-Aldrich Inc.) and 2–4 ul of extract. The PCR amplification consisted of an initial denaturing at 94 °C for four minutes, followed by 55 cycles of denaturing at 94 °C for 30 sec, annealing at 47–58 °C (depending on the primer pair) for 30 sec, and extension at 68 °C for 40 sec with a final extension period of 5 minutes at 68 °C.

The amplicons were cleaned from unincorporated primers using Exonuclease I, and Shrimp Alkaline Phosphatase (Thermo Fisher Scientific, UK). The samples were sequenced and analyzed in ABI 3100 Genetic Analyzer or Beckman-Coulter CEQT 8000 Genetic Analysis System. Sequencing was conducted on both strands.

### Authenticity criteria for the ancient DNA data

Negative controls were used during DNA extractions (every fifth sample) and in all PCR reactions. All reagents used were molecular biology grade, and when possible, were decontaminated using UV irradiation. All working areas and equipment were decontaminated using bleach, and/or UV irradiation.

All ancient samples were re-extracted and between one to two fragments were amplified. Only samples that were consistent with the results of repeated extractions and amplifications were included in the analyses.

DNA sequences have been deposited in GenBank (http://www.ncbi.nlm.nih.gov/) with accession numbers: KY765626-KY765670. 

### Phylogenetic analyses

#### Pigs

The ancient DNA sequence data were aligned manually with modern wild boar and ancient pig sequences available on GenBank. Specifically, to compare haplotype frequency in the Eastern Mediterranean by period, ancient DNA sequences mainly from East Mediterranean and Near East were added to the data obtained in this work (Fig. 3 and Supplementary Table S3). Haplotype name for each specimen was based on ANC1 terminology^25–28^.

Thirty-six ancient samples from Greece and Israel were used to construct a 3D statistical parsimony network using the script TempNet^67^ in R^68^ (Supplementary Fig. S1 and Table S1). Gaps were assigned as a fifth character. Using the Bayesian phylogenetic analysis, samples were assigned to one of three time categories: Iron Age (1,150–586 BCE), Late Bronze Age (1550–1,150 BCE) and Early to Middle Bronze Age (3500–1550 BCE).

#### Cattle

MtDNA sequences were aligned manually, and the major haplogroups T, T1, T2, T3 and T4 could be differentiated by variation within a 240-bp CR segment^11, 29^.

For comparison, ancient DNA sequences mainly from East Mediterranean and Near East were added to the data obtained in this work (Fig. 4 and Supplementary Table S3).

Twenty-three ancient samples from Greece and Israel were used to construct a 3D statistical parsimony network as described in the section above (Supplementary Fig. S2 and Table S1). Two samples from Israel (MM387, and 431) were excluded from the analysis due to missing data. Species identification of *Bos taurus taurus*/*indicus* according to the Y-chromosome was determined from the highest bit score of species listed from the BLAST search (http://blast.ncbi.nlm.nih.gov/Blast.cgi) (Supplementary Table S5).

### Morphometrical measurements

Differences in the proportions of the metacarpal bone^31^ exist among breeds of archaeological Middle Eastern, Indian, and African cattle^32^. We measured the Greatest Length (GL) and distal Breadth (BFd) of 28 archaeological specimens from Late Bronze and Iron Ages of Greece and Israel^41^ (Supplementary Table S4), and analysed their morphological distinctiveness with respect to Grigson’s (1991) dataset^32^. This was done by log-transforming the archaeological measurements and plotting them along with Grigson’s archaeological cattle against each other and by using the Linear Discriminant Analysis (LDA) module in PAST 3.11^40^.

## Electronic supplementary material

Table S1, Table S2, Table S3, Table S4, Table S5

## References

[1] Cline, E. H. *The Year Civilization Collapsed*. (Princeton: University Press, 2014).

[2] Pulak C (1998). The Uluburun shipwreck: an overview. Int J Naut Archaeol.

[3] Ward, W. A. & Sharp Joukowsky, M. *The Crisis Years*: *The 12th Century B*.*C*. *from Beyond the Danube to the Tigris*. (Kendall/Hunt Pub, 1992).

[4] Kaniewski D (2010). Late second–early first millennium BC abrupt climate changes in coastal Syria and their possible significance for the history of the Eastern Mediterranean. Quaternary Res.

[5] Langgut D, Finkelstein I, Litt T (2013). Climate and the Late Bronze Collapse: New Evidence from the Southern Levant. Tel Aviv.

[6] Yasur-Landau, A. *The Philistines and Aegean Migration at the End of the Late Bronze Age*. (Cambridge University Press, 2010).

[7] Matisoo-Smith E (2009). On the Rat Trail in Near Oceania: Applying the Commensal Model to the Question of the Lapita Colonization. Pacific Science.

[8] Pellecchia M (2007). The mystery of Etruscan origins: novel clues from Bos taurus mitochondrial DNA. P Roy Soc B-Biol Sci.

[9] Meiri, M. *et al*. *Ancient DNA and population turnover in southern Levantine pigs- signature of the Sea Peoples mgration?**Sci Rep*-*Uk* **3**, doi:Artn 3035 10.1038/Srep03035 (2013). 10.1038/srep03035 PMC3816294 24186332

[10] *Integrated Taxonomic Information System on*-*line database* http://www.itis.gov (2017).

[11] Troy CS (2001). Genetic evidence for Near-Eastern origins of European cattle. Nature.

[12] Beja-Pereira A (2006). The origin of European cattle: Evidence from modern and ancient DNA. P Natl Acad Sci USA.

[13] Reingruber A, Thissen L (2009). Depending on 14C data: chronological frameworks in the Neolithic and Chalcolithic of southeastern Europe. Radiocarbon.

[14] Chen S (2010). Zebu cattle are an exclusive legacy of the South Asia Neolithic. Mol Biol Evol.

[15] Matthews R (2002). Zebu: harbingers of doom in Bronze Age western Asia?. Antiquity.

[16] Epstein, H. *The origin of the domestic animals of Africa*. (Africana Publishing Corporation, 1971).

[17] Marshall F (1989). Rethinking the role of Bos indicus in sub-Saharan Africa. Curr Anthropol.

[18] MacHugh DE, Shriver MD, Loftus RT, Cunningham P, Bradley DG (1997). Microsatellite DNA variation and the evolution, domestication and phylogeography of taurine and zebu cattle (*Bos taurus* and *Bos indicus*). Genetics.

[19] Hanotte O (2002). African pastoralism: genetic imprints of origins and migrations. Science.

[20] Decker JE (2014). Worldwide patterns of ancestry, divergence, and admixture in domesticated cattle. PLoS Genet.

[21] Hirsch S, Schindler H (1957). The Syrian and Dutch Friesian cattle and their crosses in Israel. Ktavim.

[22] Seroussi E, Yakobson E (2010). Bovine mtDNA D-loop haplotypes exceed mutations in number despite reduced recombination: an effective alternative for identity control. Animal.

[23] Clason A (1978). Late Bronze Age- Iron Age zebu cattle in Jorden?. J Archaeol Sci.

[24] Clutton-Brock, J. *Domesticated animals from early times*. (University of Texas Press, 1981).

[25] Larson, G. *et al*. Ancient DNA, pig domestication, and the spread of the Neolithic into Europe. *Proc Natl Acad Sci USA* **104**, 15276–15281, doi:0703411104 (2007). 10.1073/pnas.0703411104 PMC1976408 17855556

[26] Larson, G. *et al*. Phylogeny and ancient DNA of *Sus* provides insights into neolithic expansion in Island Southeast Asia and Oceania. *Proc Natl Acad Sci USA* **104**, 4834–4839, doi:0607753104 (2007). 10.1073/pnas.0607753104 PMC1829225 17360400

[27] Larson, G. *et al*. Worldwide phylogeography of wild boar reveals multiple centers of pig domestication. *Science* **307**, 1618–1621, doi:307/5715/1618 (2005). 10.1126/science.1106927 15761152

[28] Ottoni, C. *et al*. Pig domestication and human-mediated dispersal in western Eurasia revealed through ancient DNA and geometric morphometrics. *Mol Biol Evol*, doi:mss261 (2012). 10.1093/molbev/mss261 PMC3603306 23180578

[29] Mannen H (2004). Independent mitochondrial origin and historical genetic differentiation in North Eastern Asian cattle. Mol Phylogenet Evol.

[30] Götherström A (2005). Cattle domestication in the Near East was followed by hybridization with aurochs bulls in Europe. P Roy Soc Lond B: Bio.

[31] Thompson, D. “AW *On growth and form*, Republished as abridged edition, JT Bonner” (Cambridge Univ. Press) (1917).

[32] Grigson C (1991). An African origin for African cattle? —some archaeological evidence. African Archaeological Review.

[33] Evin A (2015). Unravelling the complexity of domestication: a case study using morphometrics and ancient DNA analyses of archaeological pigs from Romania. Phil. Trans. R. Soc. B.

[34] Krause-Kyora, B. *et al*. Use of domesticated pigs by Mesolithic hunter-gatherers in northwestern Europe. *Nat*. *Commun* **4** (2013). 10.1038/ncomms3348 PMC3903269 23982268

[35] Vai, S. *et al*. The Biarzo case in northern Italy: is the temporal dynamic of swine mitochondrial DNA lineages in Europe related to domestication? *Sci Rep*-*Uk* **5** (2015). 10.1038/srep16514 PMC4637886 26549464

[36] Bollongino R, Edwards CJ, Alt KW, Burger J, Bradley DG (2006). Early history of European domestic cattle as revealed by ancient DNA. Biol Lett.

[37] Bonfiglio S (2012). Origin and spread of *Bos taurus*: new clues from mitochondrial genomes belonging to haplogroup T1. PLoS One.

[38] Olivieri A (2015). Mitogenomes from Egyptian Cattle Breeds: New Clues on the Origin of Haplogroup Q and the Early Spread of Bos taurus from the Near East. PLoS One.

[39] Evin A (2013). The long and winding road: identifying pig domestication through molar size and shape. J Archaeol Sci.

[40] Hammer, Ø., Harper, D. & Ryan, P. PAST: Paleontological Statistics Software Package for education and data analysis. *Palaeontolia Electronica* **4** (2001).

[41] Von den Driesch, A. A guide to the measurement of animal bones from archaeological sites: as developed by the Inst. für Palaeoanatomie, Domestikationsforschung u. Geschichte d. Tiermedizin of the Univ. of Munich. Vol. 1 (Peabody Museum Press, 1976).

[42] Maran, J. In *Between the Aegean and Baltic Seas*: *prehistory across borders* Vol. Aegaeum (eds Ioanna, Galanaki) 3–21 (Université de Liège, Belgium, 2007).

[43] Rahmstorf, L. In *Interweaving Worlds*: *Systemic Interactions in Eurasia*, *7th to 1st Millennia BC* (eds T. Wilkinson, S. Sherratt & J. Bennet) 100–119 (Oxbow Books, Oxford, 2011).

[44] Alexandri P (2012). The Balkans and the colonization of Europe: the post-glacial range expansion of the wild boar. Sus scrofa. J Biogeogr.

[45] Lega, C. *et al*. Like a pig out of water: seaborne spread of domestic pigs in Southern Italy and Sardinia during the Bronze and Iron Ages. *Heredity*, doi:http://dx.doi.org/10.1038/hdy.2016.74 (2016). 10.1038/hdy.2016.74 PMC5234479 27649620

[46] Hesse B (1990). Pig Lovers and Pig Haters: Patterns of Palestinian Pork Production. Journal of Ethnobiology.

[47] Harris, M. *The Sacred Cow and the Abominable Pig*: *riddles of food and culture* (Simon and Schuster, 1987).

[48] Redding RW (2015). The pig and the chicken in the Middle East: Modeling human subsistence behavior in the archaeological record using historical and animal husbandry data. J Archaeol Res.

[49] Haile J, Larson G, Owens K, Dobney K, Shapiro B (2010). Ancient DNA typing of archaeological pig remains corroborates historical records. J Archaeol Sci.

[50] Sapir-Hen L, Meiri M, Finkelstein I (2015). Iron Age Pigs: New Evidence on Their Origin and Role in Forming Identity Boundaries. Radiocarbon.

[51] Crabtree, P. J. Sheep, horses, swine, and kine: A zooarchaeological perspective on the Anglo-Saxon settlement of England. *J Field Archaeol* (1989).

[52] Bradley DG, Loftus RT, Cunningham P, MacHugh DE (1998). Genetics and domestic cattle origins. Evol Anthropol.

[53] Edwards CJ (2007). Mitochondrial DNA analysis shows a Near Eastern Neolithic origin for domestic cattle and no indication of domestication of European aurochs. P Roy Soc B-Biol Sci.

[54] Goren, Y., Finkelstein, I. & Na’aman, N. *Inscribed in Clay*: *Provenance Study of the Amarna Letters and other Ancient Near Eastern Texts*. Vol. 23 (Tel Aviv University 2004).

[55] Moran, W. L. *The Amarna Letters*. (Johns Hopkins Univ Pr, 1992).

[56] Martin, M. A. S. *Egyptian*-*Type Pottery in the Late Bronze Age southern Levant* (Verlag der Österreichischen Akademie der Wissenshaften, Vienna, 2011).

[57] Goldwasser O (1984). Hieratic Inscriptions from Tel Sera’in Southern Canaan. Tel Aviv.

[58] Sapir-Hen, L. I. e. In *Megiddo VI*: *the 2010–2014 Seasons*. (eds I. Finkelstein, M. Martin & M. J. Adams) (Tel Aviv, In press).

[59] Horwitz, L. & Milevski, I. The faunal evidence for socioeconomic change between the Middle and Late Bronze Age in the southern Levant. *Studies in the Archaeology of Israel and Neighboring Lands in Memory of Douglas L*. *Esse*, *Studies in Ancient Oriental Civilization* 283–306 (2001).

[60] Frumin, S., Maeir, A. M., Horwitz, L. K. & Weiss, E. Studying Ancient Anthropogenic Impacts on Current Floral Biodiversity in the Southern Levant as reflected by the Philistine Migration. *Sci Rep*-*Uk* **5** (2015). 10.1038/srep13308 PMC4642518 26304818

[61] Kroll H (1982). Kulturpflanzen von Tiryns. Archäologischer Anzeiger.

[62] Toffolo MB, Arie E, Martin MAS, Boaretto E, Finkelstein I (2014). Absolute Chronology of Megiddo, Israel, in the Late Bronze and Iron Ages: High-Resolution Radiocarbon Dating. Radiocarbon.

[63] Boaretto, E. In *Megiddo IV*: *The 1998–2002 Seasons* (eds I. Finkelstein, D. Ussishkin & B. Halpern) 550–557 (Tel Aviv, 2006).

[64] Macheridis, S. Home, refuse and reuse during the Early Helladic III to the Middle Helladic I transitional period: a social zooarchaeological study of the Asine bothroi. *Opuscula* **9**, 71–91 (2016).

[65] Yang DY, Eng B, Waye JS, Dudar JC, Saunders SR (1998). Technical note: Improved DNA extraction from ancient bones using silica-based spin columns. Am J Phys Anthropol.

[66] Loftus R (1994). Mitochondrial genetic variation in European, African and Indian cattle populations. Anim Genet.

[67] Prost S, Anderson CNK (2011). TempNet: a method to display statistical parsimony networks for heterochronous DNA sequence data. Methods in Ecology and Evolution.

[68] R Core Team R: A language and environment for statistical computing. R Foundation for Statistical Computing, Vienna, Austria. URL http://www.R-project.org/ (2013).

