# Supplementary material for: Eastern Mediterranean Mobility in the Bronze and Early Iron Ages: Inferences from Ancient DNA of Pigs and Cattle
Source: Sci Rep. 2017 Apr 6;7:701. doi: 10.1038/s41598-017-00701-y (PMC5429671; doi:10.1038/s41598-017-00701-y)
Supplement: Supplementary file 1 — Table S1, Table S2, Table S3, Table S4, Table S5 [file 41598_2017_701_MOESM1_ESM.doc]

**Eastern Mediterranean Mobility in the Bronze and Early Iron Ages: Inferences from Ancient DNA of Pigs and Cattle**

Meirav Meiri^1, 2*^, Philipp W. Stockhammer^3^, Nimrod Marom^4^, Guy Bar-Oz^4^, Lidar Sapir-Hen^1, 2^, Peggy Morgenstern^5^, Stella Macheridis^6^, Baruch Rosen^7^, Dorothée Huchon^8^, Joseph Maran^5§^, Israel Finkelstein^1§^

^1^. Institute of Archaeology, Tel Aviv University, Tel Aviv 69978, Israel

^2^. The Steinhardt Museum of Natural History, Israel National Center for Biodiversity Studies, Tel Aviv University, Tel Aviv 69978, Israel

^3^. Institute for Pre- and Protohistoric Archaeology and Archaeology of the Roman Provinces, Ludwig-Maximilians-University Munich, Schellingstraße 12, 80799 München, Germany

^4^. Zinman Institute of Archaeology, University of Haifa, Mount Carmel, Haifa 31905, Israel

^5^. Institute for Prehistory, Protohistory and Near Eastern Archaeology, University of Heidelberg, Marstallhof 4, 69117 Heidelberg, Germany

^6^. Department of Archaeology and Ancient History, Lund University, Helgonvägen 3, 223 63 Lund, Sweden

^7^. Israel Antiquities Authority, POB 180 Atlit, 30300, Israel

^8^. Department of Zoology, Tel Aviv University, Tel Aviv 69978, Israel

^§^ Contributed equally to the article

^*^ Corresponding author: Meirav Meiri, Tel Aviv University, Tel Aviv 69978 + 972 3 6409817 [meirav.meiri@gmail.com](mailto:meirav.meiri@gmail.com).

**Supplementary**

**Tables**

**Table S1**: Details of samples considered in this work.

**Table S2**: List of primer pairs used in this study.

**Table S3**: List of ancient and modern pig and cattle samples taken from GenBank for the analyses in this paper.

**Table S4**: Morphometric data.

**Table S5**: BLAST scores based on maximum identify for the two Y-chromosome SNPs (http://blast.ncbi.nlm.nih.gov/Blast.cgi)

**Supplementary Table S1**: Details of the ancient samples used in this paper.

| **Genus/ Species** | **Sample ID** | **Lab number** | **Site** | **Status (Wild/ Domestic)** | **Female/Male** | **Material** | **Period** | **Stratigraphic details** | **DNA** | **Haplotype** | **Accession numbers** |
| --- | --- | --- | --- | --- | --- | --- | --- | --- | --- | --- | --- |
| *Bos taurus* | S4 | MM501 | Asine, Greece | Domestic | N/A* | Tooth M3 | Early Helladic II-III | AS5078/Box75 | Yes | T3 | KY765658 |
| *Bos taurus* | S3 | MM503 | Asine, Greece | Domestic | N/A* | Astragal | 3655+40 uncal. (LuS10929) | AS5115/Box272 | Yes | T3 | KY765659 |
| *Bos taurus* | S15 | MM504 | Asine, Greece | Domestic | N/A* | Tooth M2 | Early Helladic II-III | AS5078/Box292 | Yes | T3 | KY765660 |
| *Bos taurus* | S14 | MM506 | Asine, Greece | Domestic | N/A* | Femur | Early Helladic II-III | AS4477/Box242 | Yes | T3 | KY765661,  KY765669,  KY765670 |
| *Bos taurus* | S11 | MM509 | Asine, Greece | Domestic | N/A* | Metatarsus | Early Helladic II | AS2199/Box269 | Yes | T3 T | KY765656 |
| *Bos taurus* | S5 | MM511 | Asine, Greece | Domestic | N/A* | Radius | 3660+40 uncal. (LuS10933) | AS5242/Box273 | Yes | T3 | KY765662 |
| *Bos taurus* | S12 | MM513 | Asine, Greece | Domestic | N/A* | Tooth M2 | Early Helladic III | AS4776/Box302 | Yes | T3 | KY765663 |
| *Bos taurus* | S13 | MM518 | Asine, Greece | Domestic | N/A* | Tooth M3 | Early Helladic III | AS4776/Box302 | Yes | T | KY765657 |
| *Bos taurus* | S16 | MM502 | Asine, Greece | Domestic | N/A* | Tooth M1 | Early HelladicII | AS4802/Box324 | No |  |  |
| *Bos taurus* | S8 | MM507 | Asine, Greece | Domestic | N/A* | Scapula | Early Helladic II | AS1228/Box89 | No |  |  |
| *Bos taurus* | S6 | MM508 | Asine, Greece | Domestic | N/A* | Humerus | Early Helladic II-III | AS2696/Box129 | No |  |  |
| *Bos taurus* | S10 | MM512 | Asine, Greece | Domestic | N/A* | Phalanx 1 | Early Helladic II | AS1283/Box89 | No |  |  |
| *Bos taurus* | S1 | MM514 | Asine, Greece | Domestic | N/A* | Metatarsus | 3724+40 uncal. (LuS10927) | AS2262/Box271 | No |  |  |
| *Bos taurus* | S2 | MM516 | Asine, Greece | Domestic | N/A* | Radius | 3670+40 uncal.(LuS10928) | AS5127/Box272 | No |  |  |
| *Bos taurus* | S9 | MM517 | Asine, Greece | Domestic | N/A* | Phalanx 1 | Early Helladic II-III | AS4768/Box302 | No |  |  |
| *Bos taurus* | S7 | MM519 | Asine, Greece | Domestic | N/A* | Radius | 3935+40 uncal. (LuS10938) | AS5201/Box261 | No |  |  |
| *Bos* | 34/87 | MM335 | Tiryns, Greece | Domestic | N/A* | Phalanx | Late Helladic IIIB final | LXII/34/87/Vig | Yes | T3 | KY765651 |
| *Bos* | 35/49 | MM347 | Tiryns, Greece | Domestic | N/A* | Carpal | Late Helladic IIIB early | LXIII/35/49/VIC | Yes | T | KY765650 |
| *Bos* | 34/99 | MM349 | Tiryns, Greece | Domestic | N/A* | Phalanx II | Late Helladic IIIB final | LXIII/34/99/VIC | Yes | T | KY765649 |
| *Bos* | 35/49 | MM367 | Tiryns, Greece | Domestic | N/A* | Phalanx | Late Helladic IIIB final | LXIII/35/49/VIA | Yes | T3 | KY765652 |
| *Bos* | 35/15 | MM370 | Tiryns, Greece | Domestic | N/A* | Phalanx | Late Helladic IIIB final | LXIII/35/15/VIC | Yes | T3 | KY765653 |
| *Bos* | 35/24 | MM373 | Tiryns, Greece | Domestic | N/A* | Humerus | Late Helladic IIIB final | LXIII/35/24/V | Yes | T3 | KY765654 |
| *Bos* | 35/38 | MM377 | Tiryns, Greece | Domestic | N/A* | Phalanx | Late Helladic IIIB final | LXIII/35/38/VIA | Yes | T3 | KY765655 |
| *Bos* | 30/90 | MM332 | Tiryns, Greece | Domestic | N/A* | Calcaneus | Late Helladic IIIC | LXVIII/30/90/X-XI | No |  |  |
| *Bos* | 35/25 | MM333 | Tiryns, Greece | Domestic | N/A* | Scapula | Late Helladic IIIB final | LXIII/35/25/VB | No |  |  |
| *Bos* | 31/26 | MM334 | Tiryns, Greece | Domestic | N/A* | Radius | Late Helladic IIIC | LXVIII/31/26/IX | No |  |  |
| *Bos* | 31/1 | MM337 | Tiryns, Greece | Domestic | N/A* | Metacarpus | Late Helladic IIIC | LXIX/31/1/X-XI | No |  |  |
| *Bos* | 30/86 | MM338 | Tiryns, Greece | Domestic | N/A* | Metacarpus | Late Helladic IIIC | LXVIII/30/86/X | No |  |  |
| *Bos* | 30/2 | MM339 | Tiryns, Greece | Domestic | N/A* | Metacarpus | Late Helladic IIIC | LXIX/30/2/X-XI | No |  |  |
| *Bos* | 31/4 | MM340 | Tiryns, Greece | Domestic | N/A* | Phalanx I and II | Late Helladic IIIC | LXVIII/31/4/X | No |  |  |
| *Bos* | 34/44 | MM342 | Tiryns, Greece | Domestic | N/A* | Phalanx | Late Helladic IIIB final | LXIII/34/44/Vid | No |  |  |
| *Bos* | 30/39 | MM343 | Tiryns, Greece | Domestic | N/A* | Metapodium | Late Helladic IIIC | LXVIII/30/39/IX | No |  |  |
| *Bos* | 30/19 | MM344 | Tiryns, Greece | Domestic | N/A* | Tibia | Late Helladic IIIC | LXVIII/30/19/IX | No |  |  |
| *Bos* | 30/74 | MM345 | Tiryns, Greece | Domestic | N/A* | Femur | Late Helladic IIIC | LXVIII/30/74/IX | No |  |  |
| *Bos* | 31/27 | MM348 | Tiryns, Greece | Domestic | N/A* | Tibia | Late Helladic IIIC | LXVIII/31/27/IX | No |  |  |
| *Bos* | 35/35 | MM350 | Tiryns, Greece | Domestic | N/A* | Talus | Late Helladic IIIB final | LXIII/35/35/V | No |  |  |
| *Bos* | 31/5 | MM352 | Tiryns, Greece | Domestic | N/A* | Mandible (no teeth) | Late Helladic IIIC | LXVIII/31/5/X | No |  |  |
| *Bos* | 31/5 | MM353 | Tiryns, Greece | Domestic | N/A* | Mandible (no teeth) | Late Helladic IIIB early | LXIII/31/5/VIB | No |  |  |
| *Bos* | 31/3 | MM354 | Tiryns, Greece | Domestic | N/A* | Phalanx II | Late Helladic IIIC | LXIX/31/3/IX | No |  |  |
| *Bos* | 35/8 | MM355 | Tiryns, Greece | Domestic | N/A* | Phalanx | Late Helladic IIIB final | LXII/35/8/VIH | No |  |  |
| *Bos* | 30/84 | MM357 | Tiryns, Greece | Domestic | N/A* | Phalanx I | Late Helladic IIIC | LXVIII/30/84/XI | No |  |  |
| *Bos* | 30/88 | MM358 | Tiryns, Greece | Domestic | N/A* | Ulna | Late Helladic IIIC | LXVIII/30/88/XI | No |  |  |
| *Bos* | 30/88 | MM359 | Tiryns, Greece | Domestic | N/A* | Femur, Phalanx II | Late Helladic IIIC | LXVIII/30/88/XI | No |  |  |
| *Bos* | 30/87 | MM360 | Tiryns, Greece | Domestic | N/A* | Mandible+ teeth | Late Helladic IIIC | LXVIII/30/87/X | No |  |  |
| *Bos* | 30/100 | MM362 | Tiryns, Greece | Domestic | N/A* | Mandible+ teeth | Late Helladic IIIC | LXVIII/30/100/X | No |  |  |
| *Bos* | 30/95 | MM363 | Tiryns, Greece | Domestic | N/A* | Tibia | Late Helladic IIIC | LXVIII/30/95/X | No |  |  |
| *Bos* | 35/48 | MM364 | Tiryns, Greece | Domestic | N/A* | Humerus | Late Helladic IIIB early | LXIII/35/48/VIC | No |  |  |
| *Bos* | 35/21 | MM365 | Tiryns, Greece | Domestic | N/A* | Phalanx II | Late Helladic IIIB final | LXIII/35/21/VIA | No |  |  |
| *Bos* | 30/16 | MM368 | Tiryns, Greece | Domestic | N/A* | Metapodium | Late Helladic IIIC | LXVIII/30/16/IX | No |  |  |
| *Bos* | 35/35 | MM369 | Tiryns, Greece | Domestic | N/A* | Phalanx | Late Helladic IIIB final | LXIII/35/35/V | No |  |  |
| *Bos* | 35/39 | MM372 | Tiryns, Greece | Domestic | N/A* | Radius | Late Helladic IIIB final | LXIII/35/39/VA | No |  |  |
| *Bos* | 30/66 | MM374 | Tiryns, Greece | Domestic | N/A* | Metatarsus | Late Helladic IIIC | LXVIII/30/66/IX | No |  |  |
| *Bos* | 30/56 | MM375 | Tiryns, Greece | Domestic | N/A* | Mandible (no teeth) | Late Helladic IIIC | LXVIII/30/56/X | No |  |  |
| *Bos* | 35/2 | MM378 | Tiryns, Greece | Domestic | N/A* | Phalanx II | Late Helladic IIIB final | LXII/35/2/VIB | No |  |  |
| *Bos* | 35/25 | MM379 | Tiryns, Greece | Domestic | N/A* | Humerus | Late Helladic IIIB final | LXIII/35/25/V | No |  |  |
| *Bos* | 35/21 | MM380 | Tiryns, Greece | Domestic | N/A* | Phalanx | Late Helladic IIIB final | LXIII/35/21/VIE | No |  |  |
| *Bos* | 1209 | MM521 | Tel Azekah, Israel | Domestic | N/A* | Distal radius | Late Bronze | s2-5/j6/251/20814 | Yes | T3 | KY765664 |
| *Bos* | 1620 | MM534 | Tel Azekah, Israel | Domestic | N/A* | Distal humerus | Late Bronze | t2-3a/c4/325/42150 | Yes | T2 | KY765665 |
| *Bos* | 246 | MM537 | Tel Azekah, Israel | Domestic | N/A* | Distal radius | Late Bronze | s2-7/j7/378/21723 | Yes | T2 | KY765666 |
| *Bos* | 1507 | MM522 | Tel Azekah, Israel | Domestic | N/A* | Distal humerus | Late Bronze | t2-3a/c4/252/41518 | No |  |  |
| *Bos* | 1952 | MM523 | Tel Azekah, Israel | Domestic | N/A* | Distal radius | Late Bronze | t2-3a/c5/258/41501 | No |  |  |
| *Bos* | 80 | MM524 | Tel Azekah, Israel | Domestic | N/A* | Proximal femur | Late Bronze | t2-3a/d6/415/42872 | No |  |  |
| *Bos* | 1606 | MM526 | Tel Azekah, Israel | Domestic | N/A* | Distal radius | Late Bronze | t2-3a/c5/258/41511 | No |  |  |
| *Bos* | 807 | MM527 | Tel Azekah, Israel | Domestic | N/A* | Proximal radius | Late Bronze | t2-3b/c5/263/41624 | No |  |  |
| *Bos* | 1706 | MM528 | Tel Azekah, Israel | Domestic | N/A* | Proximal tibia | Late Bronze | t2-3a/d5/262/41496 | No |  |  |
| *Bos* | 175 | MM529 | Tel Azekah, Israel | Domestic | N/A* | Distal tibia | Late Bronze | s2-5/j6/369/21587 | No |  |  |
| *Bos* | 1121 | MM531 | Tel Azekah, Israel | Domestic | N/A* | Distal humerus | Late Bronze | s2-4/j6/274/20874 | No |  |  |
| *Bos* | 1605 | MM532 | Tel Azekah, Israel | Domestic | N/A* | Distal tibia | Late Bronze | t2-3a/c5/258/41511 | No |  |  |
| *Bos* | 1148 | MM533 | Tel Azekah, Israel | Domestic | N/A* | Proximal radius | Late Bronze | s2-6/j6/294/21323 | No |  |  |
| *Bos* | 345 | MM536 | Tel Azekah, Israel | Domestic | N/A* | Femur | Late Bronze | s2-6/j7/370/21637 | No |  |  |
| *Bos* | 3856 | MM538 | Tel Azekah, Israel | Domestic | N/A* | Proximal femur | Late Bronze | s2-6/j6/407/22127 | No |  |  |
| *Bos* | 69 | MM539 | Tel Azekah, Israel | Domestic | N/A* | Proximal humerus | Late Bronze | t2-3a/d6/415/42872 | No |  |  |
| *Bos* | 5235 | MM387 | Tel Megiddo, Israel | Domestic | N/A* | Femur | Late Bronze Age II | 04/K8/105 | Yes | T1 | KY765647 |
| *Bos* | 96 | MM405 | Tel Megiddo, Israel | Domestic | N/A* | Ischium | Iron Age IIB | 98/H4/38 | Yes | T1 | KY765642 |
| *Bos* | 344 | MM416 | Tel Megiddo, Israel | Domestic | N/A* | Metacarpal | Late Iron Age I | 00/H5/37 | Yes | T1 | KY765643 |
| *Bos* | 757 | MM417 | Tel Megiddo, Israel | Domestic | N/A* | Femur | Late Iron Age I | 98/H5/62 | Yes | T1 | KY765644 |
| *Bos* | 269 | MM422 | Tel Megiddo, Israel | Domestic | N/A* | Femur | Late Iron Age I | 00/H5/37 | Yes | T1c | KY765645,  KY765667,  KY765668 |
| *Bos* | 110 | MM431 | Tel Megiddo, Israel | Domestic | N/A* | Cranium | Iron Age IIB | 98/H4/52 | Yes | T1c | KY765646 |
| *Bos* | 6081 | MM461 | Tel Megiddo, Israel | Domestic | N/A* | Metapod | Late Bronze Age II | 10/K8/002 | Yes | T | KY765648 |
| *Bos* | 5548 | MM384 | Tel Megiddo, Israel | Domestic | N/A* | Phalanx | Late Bronze Age II | 04/K8/081 | No |  |  |
| *Bos* | 5480 | MM385 | Tel Megiddo, Israel | Domestic | N/A* | Phalanx | Late Bronze Age II | 04/K8/119 | No |  |  |
| *Bos* | 5965 | MM386 | Tel Megiddo, Israel | Domestic | N/A* | Phalanx | Late Bronze Age II | 04/K8/099 | No |  |  |
| *Bos* | 5313 | MM389 | Tel Megiddo, Israel | Domestic | N/A* | Radius | Late Bronze Age II | 04/K8/081 | No |  |  |
| *Bos* | 5499 | MM390 | Tel Megiddo, Israel | Domestic | N/A* | Metacarpus | Late Bronze Age II | 04/K8/119 | No |  |  |
| *Bos* | 5532 | MM391 | Tel Megiddo, Israel | Domestic | N/A* | Femur | Late Bronze Age II | 06/K8/013 | No |  |  |
| *Bos* | 5387 | MM392 | Tel Megiddo, Israel | Domestic | N/A* | Metatarsus | Late Bronze Age II | 04/K8/081 | No |  |  |
| *Bos* | 5678 | MM394 | Tel Megiddo, Israel | Domestic | N/A* | Patella | Late Bronze Age II | 04/K8/079 | No |  |  |
| *Bos* | 5262 | MM395 | Tel Megiddo, Israel | Domestic | N/A* | Metacarpus | Late Bronze Age II | 04/K8/081 | No |  |  |
| *Bos* | 5902 | MM396 | Tel Megiddo, Israel | Domestic | N/A* | Calcaneus | Late Bronze Age II | 04/K8/099 | No |  |  |
| *Bos* | 5397 | MM397 | Tel Megiddo, Israel | Domestic | N/A* | Pelvis | Late Bronze Age II | 06/K8/062 | No |  |  |
| *Bos* | 5327 | MM399 | Tel Megiddo, Israel | Domestic | N/A* | Cervical | Late Bronze Age II | 04/K8/081 | No |  |  |
| *Bos* | 6041 | MM400 | Tel Megiddo, Israel | Domestic | N/A* | Tarsal | Late Bronze Age II | 04/K8/079 | No |  |  |
| *Bos* | 5504 | MM401 | Tel Megiddo, Israel | Domestic | N/A* | Metapod | Late Bronze Age II | 04/K8/081 | No |  |  |
| *Bos* | 5908 | MM402 | Tel Megiddo, Israel | Domestic | N/A* | Scapula | Late Bronze Age II | 04/K8/079 | No |  |  |
| *Bos* | 6180 | MM404 | Tel Megiddo, Israel | Domestic | N/A* | Petrosum R | Iron Age IIB | 06/K8/044 | No |  |  |
| *Bos* | 410 | MM406 | Tel Megiddo, Israel | Domestic | N/A* | Radius | Late Iron Age I | 08/H9/2 | No |  |  |
| *Bos* | 5568 | MM407 | Tel Megiddo, Israel | Domestic | N/A* | Petrosum R | Late Bronze Age II | 06/K8/062 | No |  |  |
| *Bos* | 6371 | MM409 | Tel Megiddo, Israel | Domestic | N/A* | Petrosum L | Late Bronze Age II | 06/K8/027 | No |  |  |
| *Bos* | 421 | MM410 | Tel Megiddo, Israel | Domestic | N/A* | Metatarsal | Late Iron Age I | 08/H9/38 | No |  |  |
| *Bos* | 691 | MM411 | Tel Megiddo, Israel | Domestic | N/A* | Ilium | Late Iron Age II | 98/H5/30 | No |  |  |
| *Bos* | 76 | MM412 | Tel Megiddo, Israel | Domestic | N/A* | Metacarpal | Iron Age IIB | 94/H3/76 | No |  |  |
| *Bos* | 124 | MM414 | Tel Megiddo, Israel | Domestic | N/A* | Phalanx 1 | Late Iron Age I | 06/H9/50 | No |  |  |
| *Bos* | 549 | MM415 | Tel Megiddo, Israel | Domestic | N/A* | Humerus | Late Iron Age I | 08/H9/2 | No |  |  |
| *Bos* | 685 | MM419 | Tel Megiddo, Israel | Domestic | N/A* | Tibia | Late Iron Age II | 98/H5/30 | No |  |  |
| *Bos* | 611 | MM420 | Tel Megiddo, Israel | Domestic | N/A* | Scapula | Late Iron Age II | 98/H5/68 | No |  |  |
| *Bos* | 102 | MM421 | Tel Megiddo, Israel | Domestic | N/A* | Calcaneus | Iron Age IIB | 06/H3/6 | No |  |  |
| *Bos* | 5626 | MM424 | Tel Megiddo, Israel | Domestic | N/A* | Humerus | Late Bronze Age II | 04/K8/081 | No |  |  |
| *Bos* | 134 | MM425 | Tel Megiddo, Israel | Domestic | N/A* | Femur | Iron Age IIB | 96/H3/33 | No |  |  |
| *Bos* | 442 | MM426 | Tel Megiddo, Israel | Domestic | N/A* | Humerus | Late Iron Age I | 08/H9/38 | No |  |  |
| *Bos* | 133 | MM427 | Tel Megiddo, Israel | Domestic | N/A* | Metacarpal | Iron Age IIB | 96/H3/24 | No |  |  |
| *Bos* | 6 | MM429 | Tel Megiddo, Israel | Domestic | N/A* | Humerus | Iron Age IIB | 98/H4/26 | No |  |  |
| *Bos* | 402 | MM430 | Tel Megiddo, Israel | Domestic | N/A* | Ilium | Late Iron Age I | 08/H9/2 | No |  |  |
| *Bos* | 90 | MM432 | Tel Megiddo, Israel | Domestic | N/A* | Femur | Iron Age IIB | 98/H3/49 | No |  |  |
| *Bos* | 898 | MM450 | Tel Megiddo, Israel | Domestic | N/A* | Tibia | Early Bronze Age | 06/J/081 | No |  |  |
| *Bos* | 886 | MM451 | Tel Megiddo, Israel | Domestic | N/A* | Phalanx | Early Bronze Age | 06/J/081 | No |  |  |
| *Bos* | 800 | MM452 | Tel Megiddo, Israel | Domestic | N/A* | Humerus | Early Bronze Age | 06/J/051 | No |  |  |
| *Bos* | 821 | MM453 | Tel Megiddo, Israel | Domestic | N/A* | Vertebra | Early Bronze Age | 06/J/051 | No |  |  |
| *Bos* | 943 | MM455 | Tel Megiddo, Israel | Domestic | N/A* | Pelvis | Early Bronze Age | 00/J/156 | No |  |  |
| *Bos* | 934 | MM456 | Tel Megiddo, Israel | Domestic | N/A* | Vertebra | Early Bronze Age | 00/J/156 | No |  |  |
| *Bos* | 939 | MM457 | Tel Megiddo, Israel | Domestic | N/A* | Radius | Early Bronze Age | 00/J/156 | No |  |  |
| *Bos* | 994 | MM458 | Tel Megiddo, Israel | Domestic | N/A* | Vertebra | Early Bronze Age | 06/J/065 | No |  |  |
| *Bos* | 930 | MM460 | Tel Megiddo, Israel | Domestic | N/A* | Metacarpal | Early Bronze Age | 00/J/156 | No |  |  |
| *Bos* | 6492 | MM462 | Tel Megiddo, Israel | Domestic | N/A* | Humerus | Late Bronze Age II | 06/K8/035 | No |  |  |
| *Bos* | 5350 | MM463 | Tel Megiddo, Israel | Domestic | N/A* | Radius | Late Bronze Age II | 04/K8/081 | No |  |  |
| *Bos* | 6397 | MM465 | Tel Megiddo, Israel | Domestic | N/A* | Radius | Late Bronze Age II | 06/K8/091 | No |  |  |
| *Bos* | 6073 | MM466 | Tel Megiddo, Israel | Domestic | N/A* | Metatarsus | Late Bronze Age II | 10/K8/002 | No |  |  |
| *Bos* | 6477 | MM467 | Tel Megiddo, Israel | Domestic | N/A* | Scapula | Late Bronze Age II | 06/K8/035 | No |  |  |
| *Bos* | 6113 | MM468 | Tel Megiddo, Israel | Domestic | N/A* | Scapula | Late Bronze Age II | 10/K8/002 | No |  |  |
| *Bos* | 6410 | MM470 | Tel Megiddo, Israel | Domestic | N/A* | Scapula | Late Bronze Age II | 06/K8/091 | No |  |  |
| *Bos* | 5289 | MM471 | Tel Megiddo, Israel | Domestic | N/A* | Maxilla tooth M1 | Late Bronze Age II | 04/K8/081 | No |  |  |
| *Bos* | 5186 | MM472 | Tel Megiddo, Israel | Domestic | N/A* | Maxilla tooth P4 | Late Bronze Age II | 04/K8/105 | No |  |  |
| *Bos* | 6361 | MM473 | Tel Megiddo, Israel | Domestic | N/A* | Mandible tooth M2 | Late Bronze Age II | 06/K8/013 | No |  |  |
| *Bos* | 6139 | MM475 | Tel Megiddo, Israel | Domestic | N/A* | Mandible tooth M1 | Late Bronze Age II | 06/K8/122 | No |  |  |
| *Bos* | 6731 | MM476 | Tel Megiddo, Israel | Domestic | N/A* | Radius | Late Bronze Age II | 08/K9/114 | No |  |  |
| *Bos* | 712 | MM477 | Tel Megiddo, Israel | Domestic | N/A* | Metatarsal | Iron Age II | 98/H5/30 | No |  |  |
| *Bos* | 110 | MM478 | Tel Megiddo, Israel | Domestic | N/A* | Tibia | Iron Age II | 200/H5/37 | No |  |  |

* Not Applicable by osteological methods.

| **Species** | **Sample ID** | **Lab number** | **Site** | **Status (Wild/ Domestic)** | **Female/ Male** | **Material** | **Period** | **Stratigraphic details** | **DNA** | **Haplotype** | **Accession numbers** |
| --- | --- | --- | --- | --- | --- | --- | --- | --- | --- | --- | --- |
| *Sus scrofa* | 34/88 | MM303 | Tiryns, Greece | Domestic | N/A* | Talus | Late Helladic IIIB final | LXII/34/88/VIG | Yes | Y1 | KY765634 |
| *Sus scrofa* | 35/24 | MM304 | Tiryns, Greece | Domestic | N/A* | Pelvis | Late Helladic IIIB final | LXIII/35/24/V | Yes | ANC-A | KY765635 |
| *Sus scrofa* | 35/24 | MM305 | Tiryns, Greece | Domestic | N/A* | Tibia | Late Helladic IIIB final | LXIII/35/39/VI | Yes | ANC-A | KY765637 |
| *Sus scrofa* | 30/86 | MM308 | Tiryns, Greece | Domestic | N/A* | Humerus and Femur | Late Helladic IIIC | LXVIII/30/86/XI | Yes | Y2 | KY765638 |
| *Sus scrofa* | 30/85 | MM311 | Tiryns, Greece | Domestic | N/A* | Tibia | Late Helladic IIIC | LXVIII/30/85/XI | Yes | ANC-A | KY765636 |
| *Sus scrofa* | 34/98 | MM315 | Tiryns, Greece | Domestic | N/A* | Ulna | Late Helladic IIIB final | LXIII/34/98/VI | Yes | ANC-A | KY765639 |
| *Sus scrofa* | 35/37 | MM323 | Tiryns, Greece | Domestic | N/A* | Mandible and teeth | Late Helladic IIIB early | LXIII/35/37/VIB | Yes | Y2 | KY765640 |
| *Sus scrofa* | 35/6 | MM306 | Tiryns, Greece | Domestic | N/A* | Humerus | Late Helladic IIIB final | LXIII/35/6/VIC | No |  |  |
| *Sus scrofa* | 30/73 | MM309 | Tiryns, Greece | Domestic | N/A* | Humerus | Late Helladic IIIC | LXIX/30/73/IX | No |  |  |
| *Sus scrofa* | 30/76 | MM310 | Tiryns, Greece | Domestic | N/A* | Tibia | Late Helladic IIIC | LXVIII/30/76/X | No |  |  |
| *Sus scrofa* | 35/17 | MM313 | Tiryns, Greece | Domestic | N/A* | Tibia | Late Helladic IIIB final | LXIII/35/17/IVF | No |  |  |
| *Sus scrofa* | 30/89 | MM314 | Tiryns, Greece | Domestic | N/A* | Metapodium | Late Helladic IIIC | LXVIII/30/89/XI | No |  |  |
| *Sus scrofa* | 35/38 | MM316 | Tiryns, Greece | Domestic | N/A* | Metapodium | Late Helladic IIIB final | LXIII/35/38/IVE | No |  |  |
| *Sus scrofa* | 31/4 | MM318 | Tiryns, Greece | Domestic | N/A* | Metapodium | Late Helladic IIIC | LXVIII/31/4/XI | No |  |  |
| *Sus scrofa* | 31/24 | MM319 | Tiryns, Greece | Domestic | N/A* | Tibia | Late Helladic IIIC | LXVIII/31/24/VIII | No |  |  |
| *Sus scrofa* | 30/90 | MM320 | Tiryns, Greece | Domestic | N/A* | Humerus | Late Helladic IIIC | LXVIII/30/90X-XI | No |  |  |
| *Sus scrofa* | 35/58 | MM321 | Tiryns, Greece | Domestic | N/A* | Tibia | Late Helladic IIIB early | LXIII/35/58/VIG | No |  |  |
| *Sus scrofa* | 30/31 | MM324 | Tiryns, Greece | Domestic | N/A* | Ulna | Late Helladic IIIC | LXIX/30/31/X-XI | No |  |  |
| *Sus scrofa* | 30/87 | MM325 | Tiryns, Greece | Domestic | N/A* | Mandible | Late Helladic IIIC | LXVIII/30/87/X | No |  |  |
| *Sus scrofa* | 30/94 | MM326 | Tiryns, Greece | Domestic | N/A* | Metapodium | Late Helladic IIIC | LXVIII/30/94/XI | No |  |  |
| *Sus scrofa* | 31/5N | MM328 | Tiryns, Greece | Domestic | N/A* | Mandible | Late Helladic IIIC | LXIX/31/5N/VIII | No |  |  |
| *Sus scrofa* | 30/68 | MM329 | Tiryns, Greece | Domestic | Male | Mandible | Late Helladic IIIC | LXVIII/30/68/VIII | No |  |  |
| *Sus scrofa* | 30/67 | MM330 | Tiryns, Greece | Domestic | N/A* | Mandible | Late Helladic IIIC | LXIX/30/67/VIII | No |  |  |
| *Sus scrofa* | S17 | MM480 | Asine, Greece | Probably domestic | N/A* | Radius | 3715±40 uncal. BP  (LuS 10935) | AS4659/Box 254 | Yes | Y1 | KY765628 |
| *Sus scrofa* | S19 | MM482 | Asine, Greece | Probably domestic | N/A* | Tibia | 3680±40 uncal. BP  (LuS 10941) | AS4655/Box 264 | Yes | ANC-A | KY765627 |
| *Sus scrofa* | S21 | MM484 | Asine, Greece | Probably domestic | N/A* | Scapula | 4135±45 uncal. BP  (LuS 10942) | AS2237/Box 272 | Yes | Y2 | KY765632 |
| *Sus scrofa* | S23 | MM486 | Asine, Greece | Probably domestic | N/A* | Ilium | Early Helladic III | AS4804/Box 303 | Yes | Y1 | KY765629 |
| *Sus scrofa* | S24 | MM487 | Asine, Greece | Probably domestic | N/A* | Femur | Early Helladic II-Early Helladic III | AS2696/Box 129 | Yes | ANC-C | KY765633 |
| *Sus scrofa* | S30 | MM494 | Asine, Greece | Probably domestic | N/A* | Radius | Early Helladic III | AS4629/Box 302 | Yes | Y2 | KY765631 |
| *Sus scrofa* | S31 | MM495 | Asine, Greece | Probably domestic | N/A* | Radius | Early Helladic II-Early Helladic III | AS4768/Box 252 | Yes | Y1 | KY765630 |
| *Sus scrofa* | S32 | MM496 | Asine, Greece | Probably domestic | N/A* | Humerus | Early Helladic III | AS4513/Box 129 | Yes | ANC-A | KY765626 |
| *Sus scrofa* | S18 | MM481 | Asine, Greece | Probably domestic | N/A* | Humerus | 3700±45 BP uncal.  (LuS 10940) | AS4851/Box 274 | No |  |  |
| *Sus scrofa* | S20 | MM483 | Asine, Greece | Probably domestic | N/A* | Metatarsal IV | 3595±45 BP uncal.  (LuS 10932) | AS5171/Box 272 | No |  |  |
| *Sus scrofa* | S22 | MM485 | Asine, Greece | Probably domestic | N/A* | Scapula | Early Helladic II | AS4540/Box 265 | No |  |  |
| *Sus scrofa* | S25 | MM489 | Asine, Greece | Probably domestic | N/A* | Humerus | Early Helladic III | AS4804/Box 303 | No |  |  |
| *Sus scrofa* | S26 | MM490 | Asine, Greece | Probably domestic | N/A* | Tibia | Early Helladic II-Early Helladic III | AS2373/Box 299 | No |  |  |
| *Sus scrofa* | S27 | MM491 | Asine, Greece | Probably domestic | N/A* | Humerus | Early Helladic II-Early Helladic III | AS4977/Box 303 | No |  |  |
| *Sus scrofa* | S28 | MM492 | Asine, Greece | Probably domestic | N/A* | Ulna | Early Helladic III | AS4629/Box 302 | No |  |  |
| *Sus scrofa* | S29 | MM493 | Asine, Greece | Probably domestic | N/A* | Metacarpal IV | Early Helladic II-Early Helladic III | AS4977/Box 303 | No |  |  |
| *Sus scrofa* | 135 | MM444 | Tel Megiddo, Israel | Probably domestic | N/A* | Calcaneus | Iron Age IIA | 06/H7/2 | Yes | ANC C | KY765641 |
| *Sus scrofa* | 545 | MM434 | Tel Megiddo, Israel | Probably domestic | N/A* | Mandible | Early Bronze Age | 06/J/016 | No |  |  |
| *Sus scrofa* | 5922 | MM435 | Tel Megiddo, Israel | Probably domestic | N/A* | Vertebra | Late Bronze Age | 04/K/079 | No |  |  |
| *Sus scrofa* | 6465 | MM436 | Tel Megiddo, Israel | Probably domestic | N/A* | Thoracic | Late Bronze Age | 06/K/042 | No |  |  |
| *Sus scrofa* | 6218 | MM437 | Tel Megiddo, Israel | Probably domestic | N/A* | Cervical | Late Bronze Age | 06/K/013 | No |  |  |
| *Sus scrofa* | 6362 | MM439 | Tel Megiddo, Israel | Probably domestic | N/A* | Lumbar | Late Bronze Age | 06/K/013 | No |  |  |
| *Sus scrofa* | 5268 | MM440 | Tel Megiddo, Israel | Probably domestic | N/A* | Cervical | Late Bronze Age | 04/K/081 | No |  |  |
| *Sus scrofa* | 362 | MM441 | Tel Megiddo, Israel | Probably domestic | N/A* | Femur | Late Iron Age I | 08/H9/37 | No |  |  |
| *Sus scrofa* | 165 | MM442 | Tel Megiddo, Israel | Probably domestic | N/A* | Metatarsal | Late Iron Age I | 06/H9/49 | No |  |  |
| *Sus scrofa* | 652 | MM449 | Tel Megiddo, Israel | Probably domestic | N/A* | Ischium | Late Iron Age I | 08/H9/652 | No |  |  |
| *Sus scrofa* | 139 | MM445 | Tel Megiddo, Israel | Probably domestic | N/A* | Phalanx 1 | Iron Age IIA | 06/H7/2 | No |  |  |
| *Sus scrofa* | 2 | MM446 | Tel Megiddo, Israel | Probably domestic | N/A* | Mandible | Iron Age IIB | 94/H2/78 | No |  |  |
| *Sus scrofa* | 51 | MM447 | Tel Megiddo, Israel | Probably domestic | N/A* | Maxilla | Iron Age IIB | 94/H3/48 | No |  |  |

* Not Applicable by osteological methods.

**Supplementary Table S2**: List of primer pairs used in this study for cattle

**Table S2a**: List of mtDNA primer pairs

| **Primer name** | **Sequence 5’ to 3’** | **Length (bp)** | **Reference** |
| --- | --- | --- | --- |
| AN2F 16022-16041 | TGCCCCATGCATATAAGCAA | 157 | [^1^](#_ENREF_1) |
| AN1Rev 16178-16159 | ACGCGGCATGGTAATTAAGC |
| AN1F 16159-16178 | GCTTAATTACCATGCCGCGT | 176 | [^1^](#_ENREF_1) |
| AN3Rev 16334-16314 | GAGATGTCTTATTTAAGAGGA |
| Bos1F 16021 | ATGCCCCATGCATATAAG | 91 | This study |
| Bos1R 16087 | GAATTTGACATAATGTACTATGTAC |
| Bos2F 16087 | GTACATAGTACATTATGTCAAATTC | 113 | This study |
| Bos2R 16154 | ATGGTAATTAAGCTCGTG |
| Bos3F-16164 | ATTACCATGCCGCGTGAA | 115 | This study |
| Bos3R-16259 | AAAGAACCAGATGCCTGGTA |
| U16184 | TACCATGCCGCGTGAAACCA | 129 | [^2^](#_ENREF_2) |
| L16272 | TGAGATGGCCCTGAAGAAAGAA |

**Table S2b**: List of Y-chromosome primer pairs

| **Primer name** | **Sequence 5’ to 3’** | **Length (bp)** | **Reference** |
| --- | --- | --- | --- |
| DBY1F | GTAGTAAGAGTATGCTGCT | 127 | This study |
| DBY1R | GCTGTGGTTATCTGTAAT |
| DBY7F | CCACTTTCCTAAGAATTAAGTAC | 101 | This study |
| DBY7R | ACAAAATCCCCTCTGTAA |
| ZFY4F | gaaagttcctattaaagttaaagac | 92 | This study |
| ZFY4R | CAGAAATATGAATACTAATGAACTG |

**References**

1 Troy, C. S. *et al.* Genetic evidence for Near-Eastern origins of European cattle. *Nature* **410**, 1088-1091 (2001).

2 Bollongino, R. *et al.* Modern Taurine Cattle Descended from Small Number of Near-Eastern Founders. *Mol Biol Evol* **29**, 2101-2104, doi:10.1093/molbev/mss092 (2012).

**Supplementary Table S3:** List of the ancient and modern pig and cattle samples taken from GenBank for the analyses in this paper

**Ancient *Sus scrofa***

| **Species** | **Sample ID** | **Location** | **Site** | **Status** | **Period** | **Period** | **Haplotype** | **References** |
| --- | --- | --- | --- | --- | --- | --- | --- | --- |
| *Sus scrofa* | GL402 | Romania | Icoana | Wild |  | 8930 ± 40 uncal. BP (OxA-24688) | ANC-AS2 | [^1^](#_ENREF_1) |
| *Sus scrofa* | GL404 | Romania | Icoana | Wild |  | 8950 ± 45 uncal. BP (OxA-24694) | ANC-Y2-5A | [^1^](#_ENREF_1) |
| *Sus scrofa* | GL831 | Romania | Rotbav |  | Bronze Age | 2200–1600/1500 BCE | ANC-A | [^1^](#_ENREF_1) |
| *Sus scrofa* | GL832 | Romania | Rotbav | Domestic | Bronze Age | 2200–1600/1500 BCE | ANC-A | [^1^](#_ENREF_1) |
| *Sus scrofa* | GL833 | Romania | Rotbav | Domestic | Bronze Age | 2200–1600/1500 BCE | ANC-A | [^1^](#_ENREF_1) |
| *Sus scrofa* | GL834 | Romania | Rotbav | Domestic | Bronze Age | 1300-1000 BCE | ANC-A | [^1^](#_ENREF_1) |
| *Sus scrofa* | GL835 | Romania | Rotbav | Domestic | Bronze Age | 1300-1000 BCE | ANC-A | [^1^](#_ENREF_1) |
| *Sus scrofa* | GL836 | Romania | Rotbav | Domestic | Bronze Age | 1300-1000 BCE | ANC-A | [^1^](#_ENREF_1) |
| *Sus scrofa* | GL837 | Romania | Rotbav | Domestic | Bronze Age | 1300-1000 BCE | ANC-A | [^1^](#_ENREF_1) |
| *Sus scrofa* | GL838 | Romania | Rotbav | Domestic | Bronze Age | 1300-1000 BCE | ANC-A | [^1^](#_ENREF_1) |
| *Sus scrofa* | GL839 | Romania | Rotbav | Domestic | Bronze Age | 1300-1000 BCE | ANC-A | [^1^](#_ENREF_1) |
| *Sus scrofa* | LG422 | Romania | Vitănești | Domestic | Middle Chalcolithic |  | ANC Y1 6A | [^1^](#_ENREF_1) |
| *Sus scrofa* | LG423 | Romania | Vitănești | Wild | Middle Chalcolithic |  | ANC Cside | [^1^](#_ENREF_1) |
| *Sus scrofa* | LG424 | Romania | Vitănești | Domestic | Middle Chalcolithic |  | ANC Y1 6A | [^1^](#_ENREF_1) |
| *Sus scrofa* | LG426 | Romania | Vitănești | Wild | Middle Chalcolithic |  | ANC Cside | [^1^](#_ENREF_1) |
| *Sus scrofa* | LG427 | Romania | Vitănești | Domestic | Middle Chalcolithic |  | ANC Y1 6A | [^1^](#_ENREF_1) |
| *Sus scrofa* | LG430 | Romania | Vitănești | Domestic | Middle Chalcolithic |  | ANC Y1 6A | [^1^](#_ENREF_1) |
| *Sus scrofa* | LG431 | Romania | Vitănești | Domestic | Middle Chalcolithic |  | ANC Y2 5A | [^1^](#_ENREF_1) |
| *Sus scrofa* | LG432 | Romania | Măgura-Boldul lui Moș Ivănuș | Wild | Early Neolithic |  | ANC Cside | [^1^](#_ENREF_1) |
| *Sus scrofa* | LG434 | Romania | Măgura-Boldul lui Moș Ivănuș | Wild | Early Neolithic |  | ANC Cside | [^1^](#_ENREF_1) |
| *Sus scrofa* | LG438 | Romania | Măgura Buduiasca | Domestic | Middle Neolithic |  | ANC Y1 6A | [^1^](#_ENREF_1) |
| *Sus scrofa* | LG440 | Romania | Măgura Buduiasca | Domestic | Middle Neolithic |  | ANC Cside | [^1^](#_ENREF_1) |
| *Sus scrofa* | LG660 | Romania | Chela | Domestic | Early Chalcolithic |  | ANC-Y2-5A | [^1^](#_ENREF_1) |
| *Sus scrofa* | LG802 | Romania | Luncavița | Domestic | Middle Chalcolithic |  | ANC Y1 6A | [^1^](#_ENREF_1) |
| *Sus scrofa* | LG803 | Romania | Luncavița | Domestic | Middle Chalcolithic |  | ANC Y1 6A | [^1^](#_ENREF_1) |
| *Sus scrofa* | LG804 | Romania | Luncavița | Domestic | Middle Chalcolithic |  | ANC Y1 6A | [^1^](#_ENREF_1) |
| *Sus scrofa* | LG805 | Romania | Luncavița | Domestic | Middle Chalcolithic |  | ANC Y1 6A | [^1^](#_ENREF_1) |
| *Sus scrofa* | LG806 | Romania | Luncavița | Domestic | Middle Chalcolithic |  | ANC Y1 6A | [^1^](#_ENREF_1) |
| *Sus scrofa* | LG807 | Romania | Luncavița | Domestic | Middle Chalcolithic |  | ANC Y1 6A | [^1^](#_ENREF_1) |
| *Sus scrofa* | LG808 | Romania | Luncavița | Domestic | Middle Chalcolithic |  | ANC Y1 6A | [^1^](#_ENREF_1) |
| *Sus scrofa* | LG811 | Romania | Luncavița | Domestic | Middle Chalcolithic |  | ANC A | [^1^](#_ENREF_1) |
| *Sus scrofa* | LG815 | Romania | Cascioarele |  | Early Chalcolithic |  | ANC Y1 6A | [^1^](#_ENREF_1) |
| *Sus scrofa* | LG816 | Romania | Cascioarele |  | Early Chalcolithic |  | ANC Y1 6A | [^1^](#_ENREF_1) |
| *Sus scrofa* | LG817 | Romania | Cascioarele |  | Early Chalcolithic |  | ANC Y1 6A | [^1^](#_ENREF_1) |
| *Sus scrofa* | LG819 | Romania | Bordusani | Domestic | Middle Chalcolithic |  | ANC Y1 6A | [^1^](#_ENREF_1) |
| *Sus scrofa* | LG821 | Romania | Bordusani | Domestic | Middle Chalcolithic |  | ANC Y1 6A | [^1^](#_ENREF_1) |
| *Sus scrofa* | LG822 | Romania | Bordusani | Wild | Middle Chalcolithic |  | ANC Y1 6A | [^1^](#_ENREF_1) |
| *Sus scrofa* | LG823 | Romania | Bordusani | Domestic | Middle Chalcolithic |  | ANC Y1 6A | [^1^](#_ENREF_1) |
| *Sus scrofa* | LG824 | Romania | Bordusani | Domestic | Middle Chalcolithic |  | ANC Y1 6A | [^1^](#_ENREF_1) |
| *Sus scrofa* | LG825 | Romania | Bordusani | Domestic | Middle Chalcolithic |  | ANC Y1 6A | [^1^](#_ENREF_1) |
| *Sus scrofa* | LG827 | Romania | Bordusani | Domestic | Middle Chalcolithic |  | ANC Y1 6A | [^1^](#_ENREF_1) |
| *Sus scrofa* | LG828 | Romania | Bordusani | Domestic | Middle Chalcolithic |  | ANC Y1 6A | [^1^](#_ENREF_1) |
| *Sus scrofa* | LG829 | Romania | Bordusani | Wild | Middle Chalcolithic |  | ANC Y1 6A | [^1^](#_ENREF_1) |
| *Sus scrofa* | GL1032 | Romania | Popeşti | Domestic | Bronze Age | 3000-1600 BCE | ANC-Aside | 2 |
| *Sus scrofa* | GL1034 | Romania | Popeşti | Wild? | Iron Age | 900 BCE | ANC-Aside | 2 |
| *Sus scrofa* | GL1035 | Romania | Popeşti | Domestic | Iron Age | 900 BCE | ANC-Aside | 2 |
| *Sus scrofa* | GL128 | Ukraine | South Crimea | Wild? | Mesolithic | 9th-8th Millennium BCE | ANC-Y2-5A | 2 |
| *Sus scrofa* | GL150 | Romania | Popeşti | Domestic | Bronze Age | 10th cent. BCE | ANC-Cside | 2 |
| *Sus scrofa* | GL187 | Armenia | Lake Sevan | Domestic? | Late Bronze Early Iron Ages | 13th-19th cent. BCE | ANC-Arm1T | 2 |
| *Sus scrofa* | GL188 | Armenia | Lake Sevan | Domestic? | Late Bronze Early Iron Ages | 13th-19th cent. BCE | ANC-Arm1T | 2 |
| *Sus scrofa* | GL343 | Armenia | Khatunarkh | Wild |  | 5th Millennium BCE | ANC-Arm2T | 2 |
| *Sus scrofa* | GL375 | Romania | Veterani | Undetermined | Neolithic | 5300-5000 BCE | ANC-Aside | 2 |
| *Sus scrofa* | GL376 | Armenia | Shengevit | Undetermined |  | 3rd mill-4th-early BCE | ANC-Arm2T | 2 |
| *Sus scrofa* | GL432 | Romania | Căscioarele | Domestic | Chalcolithic | Mid-5th-mid 4th Millennium BCE | ANC-Y1-6A | 2 |
| *Sus scrofa* | GL447 | Romania | Vărăşti | Undetermined | Neolithic | 5th Millennium BCE | ANC-Cside | 2 |
| *Sus scrofa* | GL484 | Syria | Chagar Bazar | Domestic | Iron Age? | 1st 1/2 2nd Millennium BCE | ANC-Arm1T | 2 |
| *Sus scrofa* | GL521 | Romania | Borduşani | Domestic | Chalcolithic | 4500-3950 BCE | ANC-Y1-6A | 2 |
| *Sus scrofa* | GL522 | Romania | Poduri | Domestic | Chalcolithic | 4500-4250 BCE | ANC-Y1-6A | 2 |
| *Sus scrofa* | GL523 | Romania | Poduri | Domestic | Chalcolithic | 4500-4250 BCE | ANC-Y1-6A | 2 |
| *Sus scrofa* | GL536/808 | Ukraine | South Crimea | Wild? | Neolithic | 7th Millennium BCE | ANC-Y2-5A | 2 |
| *Sus scrofa* | GL565 | Romania | Vărăşti | Undetermined | Neolithic | 5th Millennium BCE | ANC-Y1-6A | 2 |
| *Sus scrofa* | GL566 | Romania | Căscioarele | Wild | Chalcolithic | Mid-5th-mid 4th Millennium BCE | ANC-Cside | 2 |
| *Sus scrofa* | GL567 | Romania | Căscioarele | Wild | Chalcolithic | Mid-5th-mid 4th Millennium BCE | ANC-Cside | 2 |
| *Sus scrofa* | GL568 | Romania | Căscioarele | Domestic | Chalcolithic | Mid-5th-mid 4th Millennium BCE | ANC-Y1-6A | 2 |
| *Sus scrofa* | GL575 | Romania | Căscioarele | Wild | Chalcolithic | Mid-5th-mid 4th Millennium BCE | ANC-Cside | 2 |
| *Sus scrofa* | GL576 | Romania | Căscioarele | Domestic | Chalcolithic | Mid-5th-mid 4th Millennium BCE | ANC-Y1-6A | 2 |
| *Sus scrofa* | GL688 | Romania | Poduri | Wild | Chalcolithic | Mid-5th-mid 4th Millennium BCE | ANC-Cside | 2 |
| *Sus scrofa* | GL803 | Romania | Căscioarele | Domestic | Chalcolithic | Mid-5th-mid 4th Millennium BCE | ANC-Y1-6A | 2 |
| *Sus scrofa* | GL812 | Armenia | Beniamin | Undetermined |  | 1st. Millennium BCE | ANC-Aside | 2 |
| *Sus scrofa* | GL834 | Romania | Căscioarele | Domestic | Chalcolithic | Mid-5th-mid 4th Millennium BCE | ANC-Y1-6A | 2 |
| *Sus scrofa* | GL868 | Romania | Căscioarele | Domestic | Chalcolithic | Mid-5th-mid 4th Millennium BCE | ANC-Y1-6A | 2 |
| *Sus scrofa* | GL876 | Armenia | Tmbatir | Undetermined | Late Bronze Early Iron Ages | 13th-19th cent. BCE | ANC-Arm1T | 2 |
| *Sus scrofa* | GL891 | Armenia | Lake Sevan | Undetermined | Late Bronze Early Iron Ages | 13th-19th cent. BCE | ANC-Arm1T | 2 |
| *Sus scrofa* | GL895 | Armenia | Sevkar | Domestic |  | 7th-5th cent. BCE | ANC-Aside | 2 |
| *Sus scrofa* | GL896 | Armenia | Lake Sevan | Domestic? | Late Bronze Early Iron Ages | 13th-19th cent. BCE | ANC-Arm1T | 2 |
| *Sus scrofa* | GL900 | Romania | Măgura | Domestic | Neolithic | 5500 BCE | ANC-Y1-6A | 2 |
| *Sus scrofa* | GL903 | Romania | Măgura | Domestic | Neolithic | 5500 BCE | ANC-Y1-6A | 2 |
| *Sus scrofa* | GL906 | Romania | Bucu | Undetermined | Iron Age | 1000-800 BCE | ANC-Aside | 2 |
| *Sus scrofa* | GL986 | Romania | Schela | Wild | Mesolithic |  | ANC-Aside | 2 |
| *Sus scrofa* | GL987 | Romania | Căscioarele | Domestic? | Chalcolithic | Mid-5th-mid 4th Millennium BCE | ANC-Y1-6A | 2 |
| *Sus scrofa* | GL988 | Romania | Schela | Wild | Mesolithic | ~7000 BCE | ANC-Cside | 2 |
| *Sus scrofa* | GL992 | Armenia | Lake Sevan | Domestic? |  | 2nd Millennium BCE | ANC-Arm1T | 2 |
| *Sus scrofa* | MMP106 | Israel | Ashkelon | Domestic | Middle Bronze |  | ANC Aside | 3 |
| *Sus scrofa* | MMP112 | Israel | Tel Rehov | Undetermined | Late Iron Age IIA |  | ANC Aside | 3 |
| *Sus scrofa* | MMP117 | Israel | Tel Megiddo | Undetermined | Late Iron Age IIA |  | Y1 | 3 |
| *Sus scrofa* | MMP121 | Israel | Tel Megiddo | Undetermined | Late Bronze III |  | Y1 | 3 |
| *Sus scrofa* | MMP129 | Israel | Tel Megiddo | Undetermined | Late Bronze III |  | Y1 | 3 |
| *Sus scrofa* | MMP167 | Israel | Tel Rehov | Undetermined | Late Iron Age IIA |  | ANC Aside | 3 |
| *Sus scrofa* | MMP168 | Israel | Tel Megiddo | Undetermined | Middle Bronze I | 3430 ± 55 BP (RTK 6657) | Arm 1T | 3 |
| *Sus scrofa* | MMP175 | Israel | Tel Rehov | Undetermined | Late Iron Age IIA | 2740 ± 55 BP (RTK6659) | ANC Aside | 3 |
| *Sus scrofa* | MMP204 | Israel | Tel Megiddo | Domestic | Iron Age IIB |  | ANC Aside | 3 |
| *Sus scrofa* | MMP208 | Israel | Tel Dor | Domestic | Late Iron Age IIA |  | Arm 1T | 3 |
| *Sus scrofa* | MMP235 | Israel | Tel Dor | Undetermined | Iron Age IIB |  | ANC Aside | 3 |
| *Sus scrofa* | MMP242 | Israel | Tell es-Safi | Undetermined | Iron Age I |  | Y1 | 3 |
| *Sus scrofa* | MMP247 | Israel | Tell es-Safi | Undetermined | Iron Age I |  | Arm 1T | 3 |
| *Sus scrofa* | MMP31 | Israel | Tel Megiddo | Undetermined | Early Iron Age IIA |  | Y1 | 3 |
| *Sus scrofa* | MMP34 | Israel | Tel Megiddo | Undetermined | Early Iron Age IIA |  | Y1 | 3 |
| *Sus scrofa* | MMP58 | Israel | Tel Megiddo | Undetermined | Early Iron Age I |  | Arm 1T | 3 |
| *Sus scrofa* | MMP63 | Israel | Tel Megiddo | Undetermined | Late Bronze Age |  | Arm 1T | 3 |
| *Sus scrofa* | MMP65 | Israel | Tel Rehov | Undetermined | Late Bronze/ Iron Age I |  | Y1 | 3 |
| *Sus scrofa* | MMP66 | Israel | Tel Megiddo | Domestic | Late Iron Age I |  | Y1 | 3 |
| *Sus scrofa* | MMP91 | Israel | Tel Megiddo | Undetermined | Iron Age IIB |  | ANC Aside | 3 |
| *Sus scrofa* | BAD10 | Turkey | Bademağacı | Domestic | Early Neolithic II -3 | 7,050-6,690 BCE | ANC Y1 | 4 |
| *Sus scrofa* | BAD15 | Turkey | Bademağacı | Domestic | Early Bronze Age II | 3,100-2,000 BCE | ANC Y2 | 4 |
| *Sus scrofa* | BAD16 | Turkey | Bademağacı | Domestic | Early Bronze Age II | 3,100-2,000 BCE | ANC ARM1T | 4 |
| *Sus scrofa* | BAD17 | Turkey | Bademağacı | Domestic | Early Bronze Age II | 3,100-2,000 BCE | ANC Y1 | 4 |
| *Sus scrofa* | BAD18 | Turkey | Bademağacı | Domestic | Early Bronze Age II | 3,100-2,000 BCE | ANC ARM1T | 4 |
| *Sus scrofa* | BAD30 | Turkey | Bademağacı | Domestic | Early Neolithic II -2 | 7,000-6,000 BCE | ANC Y1 | 4 |
| *Sus scrofa* | BAD32 | Turkey | Bademağacı | Domestic | Early Neolithic II -2 | 7,000-6,000 BCE | ANC Y1 | 4 |
| *Sus scrofa* | BAD4 | Turkey | Bademağacı | Wild | Early Neolithic II -4A | 7,000-6,000 BCE | ANC Y1 | 4 |
| *Sus scrofa* | BAD47 | Turkey | Bademağacı | Domestic | Early Neolithic II -3A | 7,000-6,000 BCE | ANC Y1 | 4 |
| *Sus scrofa* | BAD5 | Turkey | Bademağacı | Domestic | Early Neolithic II -4A | 6,405-6,235 BCE | ANC Y1 | 4 |
| *Sus scrofa* | BAD52 | Turkey | Bademağacı | Domestic | Early Neolithic II -4A | 6,405-6,235 BCE | ANC ARM1T | 4 |
| *Sus scrofa* | BAD54 | Turkey | Bademağacı | Domestic | Early Neolithic II -4A | 6,405-6,235 BCE | ANC ARM1T | 4 |
| *Sus scrofa* | BAD63 | Turkey | Bademağacı | Domestic | Early Neolithic II -4B | 7,000-6,000 BCE | ANC Y1 | 4 |
| *Sus scrofa* | BAD83 | Turkey | Bademağacı | Domestic | Early Bronze Age II | 3100-2000 BCE | ANC Y1 | 4 |
| *Sus scrofa* | BAD84 | Turkey | Bademağacı | Wild | Early Bronze Age II | 3100-2000 BCE | ANC Y1 | 4 |
| *Sus scrofa* | BAD85 | Turkey | Bademağacı | Wild | Early Bronze Age II | 3100-2000 BCE | ANC Y1 | 4 |
| *Sus scrofa* | BAD86 | Turkey | Bademağacı | Wild | Early Bronze Age II | 3100-2000 BCE | ANC Y1 | 4 |
| *Sus scrofa* | BAD87 | Turkey | Bademağacı | Wild | Early Bronze Age II | 3100-2000 BCE | ANC Y1 | 4 |
| *Sus scrofa* | BAD9 | Turkey | Bademağacı | Wild | Early Neolithic II -3A | 7,000-6,000 BCE | ANC ARM1T | 4 |
| *Sus scrofa* | LG109 | Turkey | Lidar Höyük | Domestic | Iron Age | 1,291-1,055 BCE | ANC Aside | 4 |
| *Sus scrofa* | LG110 | Turkey | Lidar Höyük | Domestic | Iron Age | 1,370-1,119 BCE | ANC Aside | 4 |
| *Sus scrofa* | LG112 | Turkey | Lidar Höyük |  | Late Bronze Age |  | ANC Y2 | 4 |
| *Sus scrofa* | LG114 | Turkey | Lidar Höyük |  | Iron Age | 1,296-1,055 BCE | ANC Aside | 4 |
| *Sus scrofa* | LG115 | Turkey | Lidar Höyük |  | Middle Bronze Age III | 2,000-1,650 BCE | ANC ARM1T | 4 |
| *Sus scrofa* | LG251 | Turkey | Lidar Höyük |  | MBA II/III |  | ANC ARM1T | 4 |
| *Sus scrofa* | LG252 | Turkey | Lidar Höyük |  | Iron Age |  | ANC Y2 | 4 |
| *Sus scrofa* | LG253 | Turkey | Lidar Höyük |  | MBA II/III |  | ANC Y2 | 4 |
| *Sus scrofa* | LG254 | Turkey | Lidar Höyük |  | MBA II/III |  | ANC Y2 | 4 |
| *Sus scrofa* | LG352 | Turkey | Gordion |  | Late Bronze Age |  | ANC ARM1T | 4 |
| *Sus scrofa* | LG354 | Turkey | Gordion |  | Late Bronze Age |  | ANC Y1 | 4 |
| *Sus scrofa* | LG459 | Turkey | Çayönü |  | Neolithic |  | ANC ARM1T | 4 |
| *Sus scrofa* | LG475 | Turkey | Çamlıbel Tarlası | Domestic | Chalcolithic | 3,590-3,470 BCE | ANC ARM1T | 4 |
| *Sus scrofa* | LG476 | Turkey | Çamlıbel Tarlası | Domestic | Chalcolithic | 3,590-3,470 BCE | ANC ARM1T | 4 |
| *Sus scrofa* | LG477 | Turkey | Çamlıbel Tarlası | Domestic | Chalcolithic | 3,590-3,470 BCE | ANC Y1 | 4 |
| *Sus scrofa* | LG479 | Turkey | Çamlıbel Tarlası | Domestic | Chalcolithic | 3,590-3,470 BCE | ANC Y1 | 4 |
| *Sus scrofa* | LG480 | Turkey | Çamlıbel Tarlası | Domestic | Chalcolithic | 3,590-3,470 BCE | ANC ARM1T | 4 |
| *Sus scrofa* | LG485 | Turkey | Çamlıbel Tarlası | Domestic | Chalcolithic | 3,590-3,470 BCE | ANC Y1 | 4 |
| *Sus scrofa* | LG486 | Turkey | Çamlıbel Tarlası | Domestic | Chalcolithic | 3,590-3,470 BCE | ANC Y1 | 4 |
| *Sus scrofa* | LG488 | Turkey | Çamlıbel Tarlası | Domestic | Chalcolithic | 3,590-3,470 BCE | ANC ARM1T | 4 |
| *Sus scrofa* | LG489 | Turkey | Çamlıbel Tarlası | Domestic | Chalcolithic | 3,590-3,470 BCE | ANC Y1 | 4 |
| *Sus scrofa* | LG491 | Turkey | Çamlıbel Tarlası | Domestic | Chalcolithic | 3,590-3,470 BCE | ANC ARM1T | 4 |
| *Sus scrofa* | LG492 | Turkey | Çamlıbel Tarlası | Domestic | Chalcolithic | 3,590-3,470 BCE | ANC ARM1T | 4 |
| *Sus scrofa* | LG493 | Turkey | Çamlıbel Tarlası | Domestic | Chalcolithic | 3,590-3,470 BCE | ANC ARM1T | 4 |
| *Sus scrofa* | LG495 | Turkey | Çamlıbel Tarlası | Domestic | Chalcolithic | 3,590-3,470 BCE | ANC ARM1T | 4 |
| *Sus scrofa* | LG522 | Turkey | Sirkeli Höyük |  | Iron Age |  | ANC ARM1T | 4 |
| *Sus scrofa* | LG524 | Turkey | Sirkeli Höyük |  | Iron Age |  | ANC ARM1T | 4 |
| *Sus scrofa* | LG527 | Turkey | Sirkeli Höyük |  | Iron Age |  | ANC Y1 | 4 |
| *Sus scrofa* | LG529 | Turkey | Sirkeli Höyük |  | Iron Age |  | ANC ARM1T | 4 |
| *Sus scrofa* | M100 | Turkey | Lidar Höyük | Domestic | Iron Age | 1,200-600 BCE | ANC Aside | 4 |
| *Sus scrofa* | M101 | Turkey | Lidar Höyük | Domestic | Iron Age | 1,200-600 BCE | ANC ARM1T | 4 |
| *Sus scrofa* | M120 | Turkey | Hassek Höyük | Domestic | Chalcolithic | 6,000-3,100 BCE | ANC ARM1T | 4 |
| *Sus scrofa* | M123 | Turkey | Hassek Höyük | Domestic | Chalcolithic | 6,000-3,100 BCE | ANC ARM1T | 4 |
| *Sus scrofa* | M124 | Turkey | Hassek Höyük | Domestic | Chalcolithic | 6,000-3,100 BCE | ANC Y1 | 4 |
| *Sus scrofa* | M18 | Turkey | Hassek Höyük | Domestic | Early Bronze Age | 3,100-2,000 BCE | ANC ARM1T | 4 |
| *Sus scrofa* | M47 | Turkey | Lidar Höyük | Domestic | Middle Bronze Age II | 2,000-1,650 BCE | ANC ARM1T | 4 |
| *Sus scrofa* | M48 | Turkey | Lidar Höyük | Wild | Middle Bronze Age II | 2,000-1,650 BCE | ANC Y2 | 4 |
| *Sus scrofa* | M49 | Turkey | Lidar Höyük | Domestic | Middle Bronze Age II | 2,000-1,650 BCE | ANC ARM1T | 4 |
| *Sus scrofa* | M50 | Turkey | Lidar Höyük | Domestic | Middle Bronze Age II | 2,000-1,650 BCE | ANC ARM1T | 4 |
| *Sus scrofa* | M51 | Turkey | Lidar Höyük | Domestic | Iron Age | 1,217-1,025 BCE | ANC Aside | 4 |
| *Sus scrofa* | M52 | Turkey | Lidar Höyük | Domestic | Middle Bronze Age II | 2,000-1,650 BCE | ANC Aside | 4 |
| *Sus scrofa* | M64 | Turkey | Lidar Höyük | Domestic | Middle Bronze Age II/I | 2,000-1,650 BCE | ANC ARM1T | 4 |
| *Sus scrofa* | M68 | Turkey | Lidar Höyük | Domestic | Middle Bronze Age II/I | 2,000-1,650 BCE | ANC Y2 | 4 |
| *Sus scrofa* | M71 | Turkey | Lidar Höyük | Domestic | Middle Bronze Age II/I | 2,000-1,650 BCE | ANC ARM1T | 4 |
| *Sus scrofa* | M73 | Turkey | Lidar Höyük | Domestic | Middle Bronze Age II/I | 2,000-1,650 BCE | ANC Y2 | 4 |
| *Sus scrofa* | M74 | Turkey | Lidar Höyük | Domestic | Middle Bronze Age II/I | 2,000-1,650 BCE | ANC ARM1T | 4 |
| *Sus scrofa* | M75 | Turkey | Lidar Höyük | Domestic | Late Bronze Age | 2,000-1,650 BCE | ANC Y2 | 4 |
| *Sus scrofa* | M76 | Turkey | Lidar Höyük | Domestic | Iron Age | 1,120-900 BCE | ANC Aside | 4 |
| *Sus scrofa* | M77 | Turkey | Lidar Höyük | Domestic | Late Bronze Age | 1,650-1,200 BCE | ANC ARM1T | 4 |
| *Sus scrofa* | M78 | Turkey | Lidar Höyük | Domestic | Iron Age | 1,270-1,050 BCE | ANC Aside | 4 |
| *Sus scrofa* | M79 | Turkey | Lidar Höyük | Domestic | Late Bronze Age | 1,650-1,200 BCE | ANC ARM1T | 4 |
| *Sus scrofa* | M80 | Turkey | Lidar Höyük | Domestic | Late Bronze Age | 1,650-1,200 BCE | ANC ARM1T | 4 |
| *Sus scrofa* | M81 | Turkey | Lidar Höyük | Domestic | Late Bronze Age | 1,650-1,200 BCE | ANC ARM1T | 4 |
| *Sus scrofa* | M82 | Turkey | Lidar Höyük | Domestic | Late Bronze Age | 1,600- 1,570/1540- 1440 BCE | ANC Aside | 4 |
| *Sus scrofa* | M83 | Turkey | Lidar Höyük | Domestic | Late Bronze Age | 1,650-1,200 BCE | ANC ARM1T | 4 |
| *Sus scrofa* | M85 | Turkey | Lidar Höyük | Domestic | Early Bronze Age | 2,835-2,478 BCE | ANC ARM1T | 4 |
| *Sus scrofa* | M86 | Turkey | Lidar Höyük | Domestic | Early Bronze Age | 3,100-2,000 BCE | ANC ARM1T | 4 |
| *Sus scrofa* | M87 | Turkey | Lidar Höyük | Domestic | Early Bronze Age | 3,100-2,000 BCE | ANC ARM1T | 4 |
| *Sus scrofa* | M88 | Turkey | Lidar Höyük | Domestic | Early Bronze Age | 3,100-2,000 BCE | ANC ARM1T | 4 |
| *Sus scrofa* | M90 | Turkey | Lidar Höyük | Domestic | Early Bronze Age | 3,100-2,000 BCE | ANC ARM1T | 4 |
| *Sus scrofa* | M93 | Turkey | Lidar Höyük | Domestic | Iron Age | 1,200-600 BCE | ANC ARM1T | 4 |
| *Sus scrofa* | M94 | Turkey | Lidar Höyük | Domestic | Iron Age | 1,200-600 BCE | ANC ARM1T | 4 |
| *Sus scrofa* | M95 | Turkey | Lidar Höyük | Domestic | Iron Age | 1,200-600 BCE | ANC ARM1T | 4 |
| *Sus scrofa* | M96 | Turkey | Lidar Höyük | Domestic | Iron Age | 1,200-600 BCE | ANC Aside | 4 |
| *Sus scrofa* | M97 | Turkey | Lidar Höyük | Domestic | Iron Age | 1,200-600 BCE | ANC Aside | 4 |
| *Sus scrofa* | M98 | Turkey | Lidar Höyük | Domestic | Iron Age | 1,200-600 BCE | ANC Aside | 4 |
| *Sus scrofa* | M99 | Turkey | Lidar Höyük | Domestic | Iron Age | 1,200-600 BCE | ANC Y2 | 4 |
| *Sus scrofa* | Mal12 | Turkey | Malkayası | Wild | Chalcolithic | 5189 ± 84 bCE | ANC Y1 | 4 |
| *Sus scrofa* | Mal9 | Turkey | Malkayası | Wild | Chalcolithic | 4254 ± 61 BCE | ANC Y1 | 4 |
| *Sus scrofa* | Men4 | Turkey | Menteşe | Domestic? | Neolithic | ~6,000 BCE | ANC Y1 | 4 |
| *Sus scrofa* | Men5 | Turkey | Menteşe | Domestic? | Neolithic | ~6,000 BCE | ANC Y1 | 4 |
| *Sus scrofa* | Ulu1 | Turkey | Ulucak Höyük | Domestic | Neolithic | 6,400-5,900 BCE | ANC Y1 | 4 |
| *Sus scrofa* | Ulu20 | Turkey | Ulucak Höyük | Domestic | Neolithic | 6,400-5,900 BCE | ANC Y1 | 4 |
| *Sus scrofa* | Ulu24 | Turkey | Ulucak Höyük | Domestic | Neolithic | 6,400-5,900 BCE | ANC Y1 | 4 |
| *Sus scrofa* | Ulu27 | Turkey | Ulucak Höyük | Domestic | Neolithic | 6,400-5,900 BCE | ANC Y1 | 4 |
| *Sus scrofa* | Ulu28 | Turkey | Ulucak Höyük | Domestic | Neolithic | 6,400-5,900 BCE | ANC Y1 | 4 |
| *Sus scrofa* | Ulu48 | Turkey | Ulucak Höyük | Domestic | Neolithic | 6,400-5,900 BCE | ANC Y1 | 4 |
| *Sus scrofa* | Ulu49 | Turkey | Ulucak Höyük | Domestic | Neolithic | 6,400-5,900 BCE | ANC Y1 | 4 |

***Ancient*** Bos

| **Genus** | **Sample ID** | **Location** | **Site** | **Species** | **Date/Period** | **Haplotype** | **References** |
| --- | --- | --- | --- | --- | --- | --- | --- |
| *Bos* | D822 | Egypt | Haft Hassan Dawood | *B. taurus* | 2000-3000BP | T3 | 5 |
| *Bos* | DID3 | Georgia | Didi Gora | *?* | c. 2000-1000 BC | T/T3 | 6 |
| *Bos* | POL2 | Hungary | Polgár-Csöpszhalom | *?* | Late Neolithic | T | 6 |
| *Bos* | BER6 | Hungary | Berettyószentmárton | *?* | Neolithic | T3 | 6 |
| *Bos* | HOD4 | Hungary | Hódmezövásárhely-Gorza | *?* | Late Neolithic | T3 | 6 |
| *Bos* | POL4 | Hungary | Polgár-Csöpszhalom | *?* | Late Neolithic | T3 | 6 |
| *Bos* | POL5 | Hungary | Polgár-Csöpszhalom | *?* | Late Neolithic | T3 | 6 |
| *Bos* | SZE1 | Hungary | Szegvár-Tüzköves | *?* | Neolithic | T3 | 6 |
| *Bos* | SZE2 | Hungary | Szegvár-Tüzköves | *Aurochs* | c. 5500-5000 B.C. (Neolithic) | P | 7 |
| *Bos* | H5 | Hungary | Ecsegfalva 23 | *Aurochs* | c. 5900-5500 B.C. (Early Neolithic) | P | 7 |
| *Bos* | H3 | Hungary | Ecsegfalva 23 | *Aurochs* | c. 5900-5500 B.C. (Early Neolithic) | P | 7 |
| *Bos* | H1 | Hungary | Ecsegfalva 23 | *Aurochs* | c. 5900-5500 B.C. (Early Neolithic) | P | 7 |
| *Bos* | ALB1 | Hungary | Albertfalva | *?* | c. 2500 BC | T3 | 6 |
| *Bos* | ALB2 | Hungary | Albertfalva | *Aurochs?* | c. 2500 B.C | P | 7 |
| *Bos* | ALB4 | Hungary | Albertfalva | *Aurochs?* | c. 2500 B.C | P | 7 |
| *Bos* | ALB3 | Hungary | Albertfalva | *?* | c. 2500 B.C | T3 | 7 |
| *Bos* | MF7 | Iran | Mehrali Fars | *Bos* | 5678 cal BP (Chalcolithic) | T | 8 |
| *Bos* | QAL 4 | Iran | Qaleh Rostam | *Bos* | 8451 cal BP (Middle Neolithic ) | T | 8 |
| *Bos* | SAC 3 | Iran | Tapeh-Sang-e-Caxmaq | *Domestic Bos?* | 7373 cal BP (Late Neolithic) | T | 8 |
| *Bos* | SAC 5 | Iran | Tapeh-Sang-e-Caxmaq | *Domestic Bos?* | 7373 cal BP (Late Neolithic) | T | 8 |
| *Bos* | SAC 7 | Iran | Tapeh-Sang-e-Caxmaq | *Domestic Bos?* | 7992 cal BP (Late Neolithic ) | T | 8 |
| *Bos* | Zag 1 | Iran | Zaghe | *Bos* | 6847 cal BP (Late Neolithic) | T | 8 |
| *Bos* | JB1 | Iran | Haftavan Tappeh | *Bos* | 7927 cal BP (Middle /Late Neolithic) | T/T1 | 8 |
| *Bos* | QAB 4 | Iran | Qabrestan | *Bos* | 5442 cal BP (Chalcolithic) | T2 | 8 |
| *Bos* | TAS 1 | Iran | Tapeh-Sang-e-Caxmaq | *Domestic Bos?* | 7673 cal BP (Late Neolithic) | T2 | 8 |
| *Bos* | SAC 1 | Iran | Tapeh-Sang-e-Caxmaq | *Domestic Bos?* | 7364 cal BP (Late Neolithic) | T2 | 8 |
| *Bos* | SAC 6 | Iran | Tapeh-Sang-e-Caxmaq | *Domestic Bos?* | 7673 cal BP (Late Neolithic) | T2 | 8 |
| *Bos* | TB1 | Iran | Tappeh Borj | *Bos* | 6523 cal BP (Chalcolithic) | T3 | 8 |
| *Bos* | SAC 2 | Iran | Tapeh-Sang-e-Caxmaq | *Domestic Bos?* | 7373 cal BP (Late Neolithic ) | T3 | 8 |
| *Bos* | IRN02 | Iran | Maral Tappeh | *Aurochs?* | c. 5000 B.C | 7 | 7 |
| *Bos* | HAF2 | Iran | Haftavan Tappeh | *Bos* | 3652 cal BP (Bronze Age ) | T1 | 8 |
| *Bos* | HG1 | Iran | Hegmataneh | *Bos?* | 1800 BP (Parthian) | T3 | 8 |
| *Bos* | SVO3 | Slovakia | Svodin | *aurochs* | c. 3000 BC | P | 6 |
| *Bos* | SVO1 | Slovakia | Svodin | *B. taurus* | c. 3000 BC | T3 | 8 |
| *Bos* | SVO2 | Slovakia | Svodin | *B. taurus* | c. 3000 BC | T3 | 8 |
| *Bos* | SV03 | Slovakia | Svodin | *aurochs* | c. 3000 B.C | P | 7 |
| *Bos* | LJU1 | Slovenia | Mala Triglavca | *B. taurus* | Late Mesolithic | T3 | 6 |
| *Bos* | LJU3 | Slovenia | Mala Triglavca | *?* | 8020 +/- 50 b.p | P | 7 |
| *Bos* | Syria17 | Syria | Dja’de el Mughara | *Aurochs* | c. 8700-8300 cal. B.C. (Early Pre Pottery Neolithic B) | T3 | 8 |
| *Bos* | TB03 | Syria | Tell Brak | *B. taurus* | 4000-3000 BC | T | 9 |
| *Bos* | TB07 | Syria | Tell Brak | *B. taurus* | Late 3rd millennium | T/T3 | 8 |
| *Bos* | CH11 | Turkey | Catalhoyuk | *B. taurus* | 9000-8000BP | T/T3 | 8 |
| *Bos* | AP7 | Turkey | Asagi Pinar | *B. taurus* | c. 5250-5080 cal BC | T | 6 |
| *Bos* | AP6 | Turkey | Asagi Pinar | *B. taurus* | c. 5250-5080 cal BC | T3 | 6 |

***Modern*** Sus scrofa

| **Country, Region** | **N** | **European** | **ANC Y1** | **ANC Y2** | **ANC-Arm1T** | **ANC-Arm2T** | **Asian** | **References** |
| --- | --- | --- | --- | --- | --- | --- | --- | --- |
| Caucasus | 11 | 4 | 1 |  | 2 | 4 | -- | 2, 4, 10 |
| Egypt | 1 | -- | -- | 1 | -- | -- | -- | 2 |
| Balkan and mainland Greece | 192 | 191 | -- | -- | -- | -- | 1 | 2, 11 |
| Iran, Iraq | 28 | 4 | 2 | 10 | 1 | 2 | 9 | 2 |
| Israel | 25 | 25 | -- | -- | -- | -- | -- | 3 |
| Syria | 2 | -- | -- | -- | -- | 2 | -- | 2 |
| Tunisia | 8 | 7 | -- | 1 | -- | -- | -- | 4 |
| Turkey | 22 | 2 | 16 | 4 | -- | -- | -- | 2, 4, 10 |

**Modern *Bos taurus*** (according to Lenstra *et al*. (2014) (12) and references there in)

| **Country, Region** | **N** | **T** | **T1** | **T2** | **T3** | **References** |
| --- | --- | --- | --- | --- | --- | --- |
| Ukraine | 16 | -- | -- | 3 | 13 | 13 |
| West Anatolia | 48 | 6 | 2 | 8 | 32 | 14-15 |
| Southwest Anatolia | 76 | 20 | 4 | 20 | 32 | 14-16 |
| Iran | 6 | -- | -- | 4 | 2 | 17 |
| Egypt | 89 | 6 | 57 | 9 | 17 | 8, 14, 18 |
| Greece & Balkan | 281 | 5 | 7 | 47 | 222 | 13-14, 18-219 |
| NW Africa | 118 | -- | 116 | -- | 2 | 14, 18 |
| Israel | 149 | 4 | 30 | 27 | 88 | 22 |

**References**

1 Evin, A. *et al.* Unravelling the complexity of domestication: a case study using morphometrics and ancient DNA analyses of archaeological pigs from Romania. *Phil. Trans. R. Soc. B* **370**, 20130616 (2015).

2 Larson, G. *et al.* Ancient DNA, pig domestication, and the spread of the Neolithic into Europe. *Proc Natl Acad Sci U S A* **104**, 15276-15281, doi:0703411104 [pii]10.1073/pnas.0703411104 (2007).

3 Meiri, M. *et al.* Ancient DNA and Population Turnover in Southern Levantine Pigs- Signature of the Sea Peoples Migration? *Sci Rep-Uk* **3**, doi:Artn 303510.1038/Srep03035 (2013).

4 Ottoni, C. *et al.* Pig domestication and human-mediated dispersal in western Eurasia revealed through ancient DNA and geometric morphometrics. *Mol Biol Evol*, doi: 10.1093/molbev/mss261 (2012).

5 Bailey, J. F. *et al.* Ancient DNA suggests a recent expansion of European cattle from a diverse wild progenitor species. *P Roy Soc B-Biol Sci* **263**, 1467-1473, doi: 10.1098/rspb.1996.0214 (1996).

6 Bollongino, R., Edwards, C. J., Alt, K. W., Burger, J. & Bradley, D. G. Early history of European domestic cattle as revealed by ancient DNA. *Biol Lett-Uk* **2**, 155-159, doi:10.1098/rsbl.2005.0404 (2006).

7 Edwards, C. J. *et al.* Mitochondrial DNA analysis shows a Near Eastern Neolithic origin for domestic cattle and no indication of domestication of European aurochs. *Proc Biol Sci* **274**, 1377-1385, doi:10.1098/rspb.2007.0020 (2007).

8 Bonfiglio, S. *et al.* Origin and spread of *Bos taurus*: new clues from mitochondrial genomes belonging to haplogroup T1. *Plos One* **7**, e38601 (2012).

9 Edwards, C. J. *et al.* Ancient DNA analysis of 101 cattle remains: limits and prospects. *J Archaeol Sci* **31**, 695-710 (2004).

10 Larson, G. *et al.* Worldwide phylogeography of wild boar reveals multiple centers of pig domestication. *Science* **307**, 1618-1621, doi:10.1126/science.1106927 (2005).

11 Alexandri, P. *et al.* The Balkans and the colonization of Europe: the post-glacial range expansion of the wild boar, Sus scrofa. *J Biogeogr***39**, 713-723, doi: 10.1111/j.1365-2699.2011.02636.x (2012).

12 Lenstra, J. A. *et al.* Meta-analysis of mitochondrial DNA reveals several population bottlenecks during worldwide migrations of cattle. *Diversity* **6**, 178-187 (2014).

13 Kantanen, J. *et al.* Maternal and paternal genealogy of Eurasian taurine cattle (*Bos taurus*). *Heredity* **103**, 404-415 (2009).

14 Troy, C. S. *et al.* Genetic evidence for Near-Eastern origins of European cattle. *Nature* **410**, 1088-1091 (2001).

15 Özdemir, M. & Dogru, Ü. Determination of phylogenetic relationships of turkish native cattle breeds with other cattle breeds using mitochondrial DNA D-loop sequence polymorphism. *Asian-Austral J Anim* **22**, 955-961 (2009).

16 Magee, D. A. *Molecular Genetic Investigations of the Diversity and Origins of Old and New World Cattle Populations.* Ph.D thesis, Trinity College Dublin, (2002).

17 Achilli, A. *et al.* Mitochondrial genomes of extinct aurochs survive in domestic cattle. *Curr Biol* **18**, R157-R158 (2008).

18 Beja-Pereira, A. *et al.* The origin of European cattle: Evidence from modern and ancient DNA. *P Natl Acad Sci USA* **103**, 8113-8118, doi:10.1073/pnas.0509210103 (2006).

19 Bonfiglio, S. *et al.* The enigmatic origin of bovine mtDNA haplogroup R: sporadic interbreeding or an independent event of *Bos primigenius* domestication in Italy? *Plos One* **5**, e15760 (2010).

20 Radoslavov, G., Hristov, P., Neov, B. & Teofanova, D. Mitochondrial diversity in Bulgarian native cow breeds. GenBank 2013. *KF373013–KF373030*.

21 Hristov, P., Teofanova, D., Neov, B., Shivachev, B. & Radoslavov, G. Mitochondrial diversity in autochthonous cattle breeds from the Balkan Peninsula. *Czech J Anim Sci* **60**, 311-318 (2015).

22 Seroussi, E. & Yakobson, E. Bovine mtDNA D-loop haplotypes exceed mutations in number despite reduced recombination: an effective alternative for identity control. *animal* **4**, 1818-1822 (2010).

**Supplementary Table S4:** Morphometric data

| **Data point** | **Group** | **Log (Greatest Length ) (mm)** | **Log (distal Breadth) (mm)** |
| --- | --- | --- | --- |
| Grigson (1991:Fig. 6) ^1^ | India | 2.29 | 1.69 |
| Grigson (1991:Fig. 6) ^1^ | India | 2.31 | 1.74 |
| Grigson (1991:Fig. 6) ^1^ | India | 2.32 | 1.74 |
| Grigson (1991:Fig. 6) ^1^ | India | 2.33 | 1.73 |
| Grigson (1991:Fig. 6) ^1^ | India | 2.34 | 1.76 |
| Grigson (1991:Fig. 6) ^1^ | India | 2.35 | 1.81 |
| Grigson (1991:Fig. 6) ^1^ | India | 2.37 | 1.81 |
| Grigson (1991:Fig. 6) ^1^ | India | 2.37 | 1.85 |
| Grigson (1991:Fig. 6) ^1^ | India | 2.37 | 1.86 |
| Grigson (1991:Fig. 6) ^1^ | Africa | 2.31 | 1.72 |
| Grigson (1991:Fig. 6) ^1^ | Africa | 2.32 | 1.71 |
| Grigson (1991:Fig. 6) ^1^ | Africa | 2.33 | 1.74 |
| Grigson (1991:Fig. 6) ^1^ | Africa | 2.32 | 1.79 |
| Grigson (1991:Fig. 6) ^1^ | Africa | 2.33 | 1.74 |
| Grigson (1991:Fig. 6) ^1^ | Africa | 2.33 | 1.75 |
| Grigson (1991:Fig. 6) ^1^ | Africa | 2.34 | 1.77 |
| Grigson (1991:Fig. 6) ^1^ | Africa | 2.34 | 1.81 |
| Grigson (1991:Fig. 6) ^1^ | Africa | 2.34 | 1.73 |
| Grigson (1991:Fig. 6) ^1^ | Africa | 2.36 | 1.78 |
| Grigson (1991:Fig. 6) ^1^ | Africa | 2.37 | 1.85 |
| Grigson (1991:Fig. 6) ^1^ | Africa | 2.38 | 1.84 |
| Grigson (1991:Fig. 6) ^1^ | Middle East | 2.23 | 1.69 |
| Grigson (1991:Fig. 6) ^1^ | Middle East | 2.26 | 1.7 |
| Grigson (1991:Fig. 6) ^1^ | Middle East | 2.26 | 1.81 |
| Grigson (1991:Fig. 6) ^1^ | Middle East | 2.28 | 1.72 |
| Grigson (1991:Fig. 6) ^1^ | Middle East | 2.27 | 1.78 |
| Grigson (1991:Fig. 6) ^1^ | Middle East | 2.275 | 1.72 |
| Grigson (1991:Fig. 6) ^1^ | Middle East | 2.285 | 1.72 |
| Grigson (1991:Fig. 6) ^1^ | Middle East | 2.285 | 1.765 |
| Grigson (1991:Fig. 6) ^1^ | Middle East | 2.28 | 1.85 |
| Grigson (1991:Fig. 6) ^1^ | Middle East | 2.285 | 1.75 |
| Grigson (1991:Fig. 6) ^1^ | Middle East | 2.285 | 1.765 |
| Grigson (1991:Fig. 6) ^1^ | Middle East | 2.285 | 1.775 |
| Grigson (1991:Fig. 6) ^1^ | Middle East | 2.29 | 1.75 |
| Grigson (1991:Fig. 6) ^1^ | Middle East | 2.29 | 1.85 |
| Grigson (1991:Fig. 6) ^1^ | Middle East | 2.29 | 1.71 |
| Grigson (1991:Fig. 6) ^1^ | Middle East | 2.3 | 1.715 |
| Grigson (1991:Fig. 6) ^1^ | Middle East | 2.3 | 1.76 |
| Grigson (1991:Fig. 6) ^1^ | Middle East | 2.305 | 1.755 |
| Grigson (1991:Fig. 6) ^1^ | Middle East | 2.31 | 1.735 |
| Grigson (1991:Fig. 6) ^1^ | Middle East | 2.31 | 1.77 |
| Grigson (1991:Fig. 6) ^1^ | Middle East | 2.31 | 1.84 |
| Grigson (1991:Fig. 6) ^1^ | Middle East | 2.32 | 1.77 |
| Grigson (1991:Fig. 6) ^1^ | Middle East | 2.31 | 1.805 |
| Grigson (1991:Fig. 6) ^1^ | Middle East | 2.32 | 1.825 |
| Grigson (1991:Fig. 6) ^1^ | Middle East | 2.325 | 1.815 |
| Grigson (1991:Fig. 6) ^1^ | Middle East | 2.33 | 1.8 |
| Grigson (1991:Fig. 6) ^1^ | Middle East | 2.33 | 1.82 |
| Grigson (1991:Fig. 6) ^1^ | Middle East | 2.335 | 1.835 |
| Grigson (1991:Fig. 6) ^1^ | Middle East | 2.34 | 1.84 |
| Grigson (1991:Fig. 6) ^1^ | Middle East | 2.345 | 1.875 |
| Abel Beth Maacha (Iron Age I) | Israel | 2.27 | 1.72 |
| Abel Beth Maacha (Iron Age I) | Israel | 2.29 | 1.72 |
| Tel Rehov (Iron Age II) | Israel | 2.26 | 1.78 |
| Tel Rehov (Iron Age II) | Israel | 2.26 | 1.67 |
| Tel Qashish (Iron Age) | Israel | 2.3 | 1.74 |
| Tel Qashish (Iron Age) | Israel | 2.27 | 1.7 |
| Tel Dor (Iron Age II) | Israel | 2.29 | 1.71 |
| Tel Dor (Iron Age II) | Israel | 2.27 | 1.7 |
| Tel Dor (Iron Age II) | Israel | 2.27 | 1.69 |
| Tel Dor (Iron Age II) | Israel | 2.29 | 1.77 |
| Bet Shemesh (Late Bronze) | Israel | 2.29 | 1.7 |
| Azeqa (Late Bronze) | Israel | 2.26 | 1.69 |
| Lachish 1 (Iron Age) | Israel | 2.29 | 1.72 |
| Lachish 2 (Iron Age) | Israel | 2.3 | 1.74 |
| Tel Dor (Iron Age I) | Israel | 2.29 | 1.79 |
| Tel Dor (Iron Age I) | Israel | 2.29 | 1.78 |
| Tel Dor (Iron Age I) | Israel | 2.31 | 1.8 |
| Megiddo (Iron Age IIA) | Israel | 2.25 | 1.68 |
| Tiryns (Late Bronze) ^2^ | Greece | 2.24 | 1.76 |
| Tiryns (Late Bronze) ^2^ | Greece | 2.29 | 1.8 |
| Tiryns (Late Bronze) ^2^ | Greece | 2.22 | 1.7 |
| Tiryns (Late Bronze) ^2^ | Greece | 2.26 | 1.75 |
| Tiryns (Late Bronze) ^2^ | Greece | 2.27 | 1.77 |
| Tiryns (Late Bronze) ^2^ | Greece | 2.27 | 1.77 |
| Tiryns (Late Bronze) ^2^ | Greece | 2.25 | 1.78 |
| Tiryns (Late Bronze) ^2^ | Greece | 2.21 | 1.68 |
| Tiryns (Late Bronze) ^2^ | Greece | 2.23 | 1.71 |
| Tel Azor (Iron Age) | Israel | 2.28 | 1.74 |

**References**

1. Grigson, C. An African origin for African cattle?—some archaeological evidence. *African Archaeological Review* **9**, 119-144 (1991).

2. von den Driesch, A. & Boessneck, J. Die Tierreste von der Mykenischen Burg Tiryns bei Nauplion/Peloponnes (Tiryns. Forschungen und Berichte Band XI): 87-164. *Mainz am Rhein: Verlag Philipp von Zabern* (1990).

**Supplementary Table S5**: BLAST scores based on maximum identify for the two Y-chromosome SNPs (<http://blast.ncbi.nlm.nih.gov/Blast.cgi>)

| Query sequence name | Marker DBY7  (Maximum identify, top hits, and accession numbers) | Marker ZFY4R  (Maximum identify, top hits, and accession numbers) |
| --- | --- | --- |
| MM422 | 100% *Bos taurus indicus* (EU547262)  98% Bos taurus taurus (EU547264), Bos grunniens (JX503535) | 100% Bos taurus indicus (EU547266)  98% Bos taurus taurus (AH011261), Bos grunniens (JX503534) |
| MM506 | 100% *Bos taurus taurus* (EU547264)*, Bos grunniens* (JX503535)  98% Bos taurus indicus (EU547262) | 100% Bos taurus taurus (AH011261), Bos grunniens (JX503534)  98% Bos taurus indicus (EU547266) |
